# Qualitative and Quantitative Protein Complex Prediction Through Proteome-Wide Simulations

**DOI:** 10.1371/journal.pcbi.1004424

**Published:** 2015-10-22

**Authors:** Simone Rizzetto, Corrado Priami, Attila Csikász-Nagy

**Affiliations:** 1 The Microsoft Research-University of Trento Centre for Computational Systems Biology, Rovereto, Italy; 2 Department of Mathematics, University of Trento, Povo (TN), Italy; 3 Department of Computational Biology, Research and Innovation Centre, Fondazione Edmund Mach, San Michele all'Adige, Italy; 4 Randall Division of Cell and Molecular Biophysics and Institute for Mathematical and Molecular Biomedicine, King's College London, London, United Kingdom; Wake Forest University, UNITED STATES

## Abstract

Despite recent progress in proteomics most protein complexes are still unknown. Identification of these complexes will help us understand cellular regulatory mechanisms and support development of new drugs. Therefore it is really important to establish detailed information about the composition and the abundance of protein complexes but existing algorithms can only give qualitative predictions. Herein, we propose a new approach based on stochastic simulations of protein complex formation that integrates multi-source data—such as protein abundances, domain-domain interactions and functional annotations—to predict alternative forms of protein complexes together with their abundances. This method, called SiComPre (Simulation based Complex Prediction), achieves better qualitative prediction of yeast and human protein complexes than existing methods and is the first to predict protein complex abundances. Furthermore, we show that SiComPre can be used to predict complexome changes upon drug treatment with the example of bortezomib. SiComPre is the first method to produce quantitative predictions on the abundance of molecular complexes while performing the best qualitative predictions. With new data on tissue specific protein complexes becoming available SiComPre will be able to predict qualitative and quantitative differences in the complexome in various tissue types and under various conditions.

## Author Summary

Most proteins are biologically active only when part of a complex with other proteins of the same or other type. Hence, to unravel biological functions of proteins, it is important to identify the type of complexes they can form. Multiple copies of each protein are present in cells and some of these could be involved in multiple complexes, thus it is a challenging task to identify protein complex compositions and abundances of all possible complexes. In this article we propose an integrative computational approach able to predict protein complexes from existing data sources on protein-protein and domain-domain interactions and protein abundances. By merging this information we built a computational model of all proteins and their dynamic interactions. Using cell-specific data we performed multiple stochastic simulations to predict protein complexes specific to budding yeast and human cells. Our predictions on protein complex compositions are consistent with a manually curated dataset and, for the first time, provide an approximation of their abundances. Our simulations can also predict how perturbations by a drug can influence the composition and abundance of protein complexes.

## Introduction

Mass-spectrometry (MS) techniques solved many fundamental issues in the identification of protein complexes [1–3] and other high-throughput techniques allowed the identification of Protein-Protein Interactions (PPI) and Domain-Domain Interactions (DDI), which paved the way for computational methods to predict protein complexes [4, 5]. Validation of these computational approaches is based on the existence of data on detected protein complexes in the budding yeast *Saccharomyces cerevisiae* [6–9] and on initial data on *Homo sapiens* [10, 11]. Unfortunately, all existing complex prediction methods produce only qualitative results even though protein complexes are formed dynamically and in various amounts throughout cell life. Note also that proteins with low abundance and with many possible binding partners might limit complex formation [12]. Therefore, it is crucial to predict the quantity of protein complexes.

Graph theory algorithms to predict clusters that match protein complexes [13–15] or replicate structural properties of protein complexes retrieved from in vitro experiments have been applied [14]. Recently a new clustering algorithm [15] considerably improved predictions by allowing the overlapping of protein complexes with a reference protein-protein interaction network (PPIN). Herein, we propose a method which simulates dynamic complex formation that relies on complementary binding sites of proteins and that considers absolute protein levels [16, 17] as initial number of molecular entities, in order to predict both the existence of a particular complex and its quantity. Protein binding sites correspond to domains and merging DDI and PPI data we built a proteome-wide model of all interactions in *S*. *cerevisiae* and *H*. *sapiens*. We consider DDIs only between proteins with a corresponding PPI, but the same domain of a given protein can be bound by multiple proteins with matching DDI and PPI leading to competition for binding sites and limiting formation of unrealistically large complexes. This ensures that proteins with high number of possible interactors do not interact with all possible partners at the same time and limits the size of such complexes [18]. The method was tested on protein complex prediction and it produced both exceptional qualitative results and the first quantitative prediction on protein complexes. We have also examined how the addition of a drug (the proteasome inhibitor, bortezomib in this case) influences the complexome in a qualitative and quantitative fashion. This served as a proof of concept towards protein complex prediction based drug design [19, 20].

## Materials and Methods

### Simulation settings

Our approach considered protein domains, retrieved with SMART [21], together with their corresponding DDI [22] and PPI [23]. We ran stochastic simulations for a reaction-diffusion system where multiple instances of proteins (corresponding to the square root of detected protein levels [16]) move and interact randomly on a two-dimensional logical space inspired by the Gillespie MultiParticle algorithm (GMP) [24]. We considered the square root of the absolute protein expression levels and a 2D simulation environment to reduce the computational cost, while keeping the possibilities for all proteins to meet any other protein in a reasonable time (S1 Text). In classic Gillespie algorithm [25], space is not explicity considered and the diffusion of molecules is assumed to be only a limiting factor on the reaction rates. It is absolutely important to consider space when simulating protein complex formation since closely located proteins or proteins that already participate in the same complex should have higher probability to bind with each other. Therefore, simulation algorithms that do not consider space cannot capture the right behavior of complexation and decomplexation of proteins. We considered a two-dimensional simulation space instead of the real three dimensional structure (3D) of the cell, because a well-discretized 2D space is already enough to reduce the probability of distant proteins to bind each other. Consideration of the real 3D structure of the cell would make the simulations more realistic, but the increase of computational costs would be outweighed by the benefits of considering diffusion in the third dimension.

### Protein binding sites and molecular crowding

**Fig 1:**
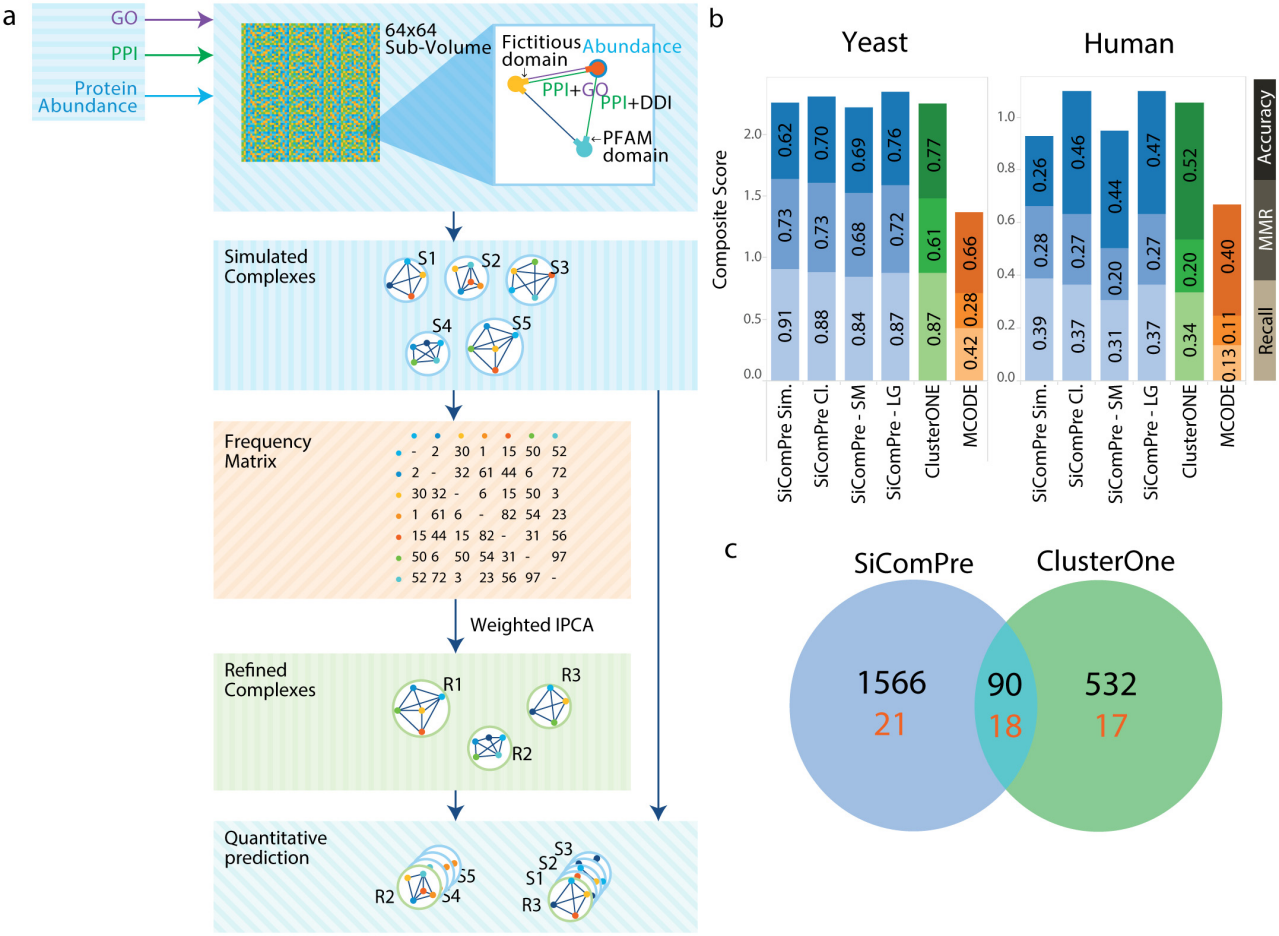
The SiComPre algorithm and its performance in predicting protein complexes. a, The SiComPre algorithm. Each protein is represented by a different colour node, edges show their interactions through domains (either known DDI or predicted from PPI by common GO annotations). For each protein pairs that appear in a simulated complex (S1–S5 second panel) the corresponding frequency matrix element increases by one. Refined complexes are formed by clustering this matrix (more details in the Online Methods). b, Performance of qualitative prediction of budding yeast and human protein complexes by SiComPre are compared with results of two of the most representative methods [13, 15]. SiComPre SIM—all simulated complexes considered, SiComPre CL—all refined complexes considered, SiComPre-LG—after low abundance large RCs dropped and SiComPre-SM—after low abundance small RCs dropped (see Methods for details and S1 Text to see how these performed in the alternative f-score system). c, Prediction of human protein complexes of SiComPre compared to predictions of ClusterOne. In blue we report the set of complexes predicted by SiComPre, in green those predicted by ClusterOne. The red numbers indicate the matched complexes in the CORUM dataset after the process of removing redundant complexes.

We divided the simulation space into square lattices, called sub-volumes (SV), where proteins are diffused randomly between neighbour lattices at discrete time steps. As a further simplification, we used the same diffusion rate for each protein (this could be improved with proteome wide data on diffusion rates becoming available). Proteins are represented as complex objects with binding sites corresponding to domains similarly to the BlenX modelling language [26]. Complementary binding sites can interact to form complexes and their bonds can break and lead to sub-complexes (Fig 1A). The inclusion of domains as binding sites allows competition between proteins for a given binding site. As a result, in our simulations two proteins cannot bind to the same domain, which can have an impact on the formation of protein complexes when two competing proteins are present in different abundances. Due to molecular crowding and to the stochastic nature of interactions, the simulations might lead to results that depend on the initial position of each molecule. To reduce this effect, we consider multiple simulation runs with random initial conditions and protein complexes are extracted from multiple time points of the simulation. The reported results came from two simulations with different initial localizations of proteins in space. We collected the list of simulated protein complexes at two separate simulation time points (four points in total). We found that more than two simulation runs do not increase the overall performance of the method: more complexes can be found, but prediction accuracy decreases (S1 Fig). This finding could be explained by the combination of the robustness of our simulation based method (See S1 Text for details) and the limited information on protein complexes reported in reference datasets [6, 9]. Indeed a single simulation is enough to identify 90% of reference complexes, and two more simulations increase the percentage only to 91% (S1 Text). A complication comes from discrepancies between PPIs and DDIs data: only 34% of protein pairs involved in a PPI have a corresponding DDI between identified domains of the involved proteins. To enable interactions between proteins involved in a PPI, but missing proper DDI pair, we added fictitious interacting domains. Various strategies were tested (S1 Text) and the best solution was found to be the addition of fictitious domains to a pair of interacting proteins only if they are involved in the same biological function, according to MIPS [6]. Therefore, we consider DDI information between a pair of proteins only if a corresponding PPI exists. This step increased the ratio of PPIs with corresponding DDI to 84%. Our yeast model consists of 1474 proteins and the model based on data from humans contains 2342 proteins (Table 1). The presence of fictitious domains in some of the predicted complexes cannot be used to reject a prediction but if the fraction of fictitious domains in a complex is low it strengthen the predictions, as it is based on known domain-domain interactions. The computation intensive simulations were run on GPUs supporting CUDA (details in S1 Text).

**Table 1:** Protein coverage summary and number of predicted complexes.

	Input				Final Model		Predicted Complexes	
	Proteins in PPI dataset	Interactions in PPI dataset	Proteins with Abundance	Proteins with Domains	Proteins in the model	Interactions in the model	Matching Complexes	New Complexes
Yeast	1622	9022	6234	5374	1474	7618	409	248
Human	3006	13992	7309	2285	2342	9395	268	890

PPI datasets used in this study contain 1622 and 3006 proteins for yeast and human respectively [23]. In yeast, all of these proteins can be found in the abundance datasets [3, 16], while in human only 88% of proteins in the PPI network have data on protein abundances [10]. More information is known about domains in yeast than in human [21]. The final yeast model contains 84% of the interactions and 91% of proteins from the initial PPI network. The human model contains 67% of the interactions and 76% of the proteins of the PPI network (Table 1). The missing proteins and interactions are due to the lack of DDI interactions or shared GO function between two proteins of the same interaction (S1 Text for additional details about model generation). The whole pipeline generated 657 complexes in yeast from which 248 do not match any known complexes [9]. From the human data we predicted 1158 complexes and 890 of these cannot be associated to any CORUM complex.

### Prediction refinements

The simulation produces a list of complexes together with their structure (Simulated Complexes, SCs). Many SCs are constituted of similar set of proteins (S1 Text). To quantify how many overlapping complexes we detected, we apply a refinement process based on a frequency matrix where each element represents how many times a pair of proteins interacted in SCs [14]. Clustering this matrix generates the refined complexes (RCs) and the total number of SCs associated with a RC gives the quantity of that RC. Abundance of a RC is used to further increase the performance of SiComPre by dropping complexes below a threshold abundance and above or below a threshold size (details in S1 Text). An overview of the algorithm that we named SiComPre for Simulation based Complex Prediction is shown in Fig 1A.

## Results

### Qualitative predictions

The quality check of predicted protein complexes was done by comparing them with experimentally detected complexes from various sources [6, 9]. We used well accepted scoring methods to compare predicted and experimentally detected complexes: *recall* gives the fraction of properly predicted complexes [13]; *maximal matching ratio (MMR)* measures the ratio of one-to-one matching between reference and predicted complexes [15] and the geometric *accuracy* is a function of proper and improper protein associations to complexes [27] (S1 Text). A sum of these scores leads to a global measure (*composite score*) quantifying the performance of the prediction [15]. Qualitatively similar results measured by an alternative scoring system (called *f-score*) [13] are discussed in S1 Text. The scores of SiComPre and existing algorithms for budding yeast are presented in Fig 1B. We also show how SiComPre scores change at various steps of our prediction method (Fig 1A): stopping the process at the simulated complexes (SiComPre SIM) after clustering (SiComPre CL). The *composite score* of SiComPre CL are equal or higher than any previous methods (Fig 1B and S1 Text). Since we can quantify the abundance of each predicted complexes, we could evaluate how SiComPre performs when low abundance complexes are dropped from the list. Two alternative versions were tried by dropping low abundance large size complexes (SiComPre-LG) or low abundance small size complexes (SiComPre-SM) (S1 Text) and found that SiComPre-LG outperforms all other methods on the basis of the *composite score* and SiComPre-SM works the best in the alternative *f-score* prediction measurement system (S1 Text). This highlights that both scoring systems differentially penalize wrong predictions of large and small complexes but SiComPre still performs well in both systems. Other protein complex prediction methods could be investigated, but ClusterOne was already proved to perform better than each of these [15]. Note also that the clustering and dropping of low abundance complexes slightly reduces the *recall*, but increases *accuracy*, thus the direct simulation results could be used to predict higher fraction of complexes (0.9055 instead of 0.874), but with lower composite score (2.2573 instead of 2.3472). S1 Text show that use of alternative databases with somewhat differing PPIs [23] or changes in initial data or in prediction scoring [13] do not change the high performance of all versions of SiComPre. The full process generated 657 protein complexes. 248 of these have an overlap score ≤ 0.25, thus these are considered as newly predicted protein complexes (Tables 1 and S1). We also tested whether the consideration of protein abundances can effectively improve the qualitative predictions of SiComPre. We ran simulations where the abundance of all proteins were set to the average (7491 molecules) of all protein abundances in the input dataset [16]. The qualitative results show a decrement of the composite score compared to simulation that uses actual protein abundances: composite score after the simulation step is 2.16 compared to 2.26 of using actual protein abundances (S2 Fig). Therefore, incorporating protein abundance information is improving qualitative protein complex predictions.

### Quantitative predictions

SiComPre has the best scores available in the literature and it can also predict the abundance of protein complexes by counting the number of SCs overlapping a RC that can then be associated with experimentally identified complexes. S1 Table lists all our predicted complexes with their predicted abundances and their associated best matching reference complexes. Comprehensive validation of these quantitative predictions is impossible at the moment since we lack a reference dataset on protein complex abundances. However, some of the predictions can be validated according complex abundances published in the literature [28–33] (Table 2). For instance we predicted ~2,200 copies of RNA polymerase complex I, ~3,900 RNA polymerase complex II and 144 RNA polymerase complex III. Data is available for the RNA polymerase II holoenzyme in haploid yeast in the range of 2,000 to 4,000 complexes [30]. The proportion of polymerases is maintained with respect to *Mus Musculus*, where their quantification is ~30,000, ~60,000 and ~3,000 respectively [29]. Approximately 50,000 copies of ribosomes were detected in our simulations that were based on the initial protein abundance data of 7.0 × 10^4^ on average for all ribosomal subunits. In logarithmic growing yeast cells the estimated ribosome number is 187,000 ± 56,000 ribosomes [28], but this calculation was based on the average concentration of 3.15 × 10^5^ subunits per cell and assumes that all ribosomal rRNAs are involved in ribosome formation, thus our quantitative prediction could be realistic.

**Table 2:** Summary of quantitative predictions of protein complex abundances in yeast.

Protein complex	Experimental abundance	SiComPre predicted abundance	Average of constitutive subunit abundances	Experimental Evidences
Ribosome	187,000	50,000	53,331	An average of 187,000 copies has been estimated [28].
RNA polymerase I	1,500	2,200	5,031	Assuming the proportion of RNA polymerases in Mus Musculus[29] is maintained in yeast, the abundance is the half of that of RNA polymerase II.
RNA polymerase II	3,000	3,900	4,375	An average of 3,000 complexes has been estimated in Yeast[30].
RNA polymerase III	150	144	3,352	Similary for RNA polymerase I, approximated to be 1/20 of that of RNA polymerase II
Nuclear Pore Complex	200	462	2,120	An average of 200 NPC has been estimated[31].
Eisosome	75	80,372	109,500	It is the average of Eisosomes in yeast[32].
Nucleosome	57,000	90,300	267,640	The estimated number of nucleosomes in yeast[33].
Anaphase-Promoting Complex	3,000	1,406	494,2	There are about 1000–5000 APC per cell in yeast[32].

Only a few protein complex abundances are available in the literature. We summarized these, providing also a short explanation of how these were estimated. We compared SiComPre predictions against the trivial method of predicting protein complex abundances using the average of the abundance of the constitutive subunits. SiComPre predictions show a better agreement to experimental data compared to predictions based on the protein abundance averages. The predicted abundances were rescaled by squaring the value predicted from the simulation to re-establish the linear dependence between SiComPre predictions and experimental data.

**Fig 2:**
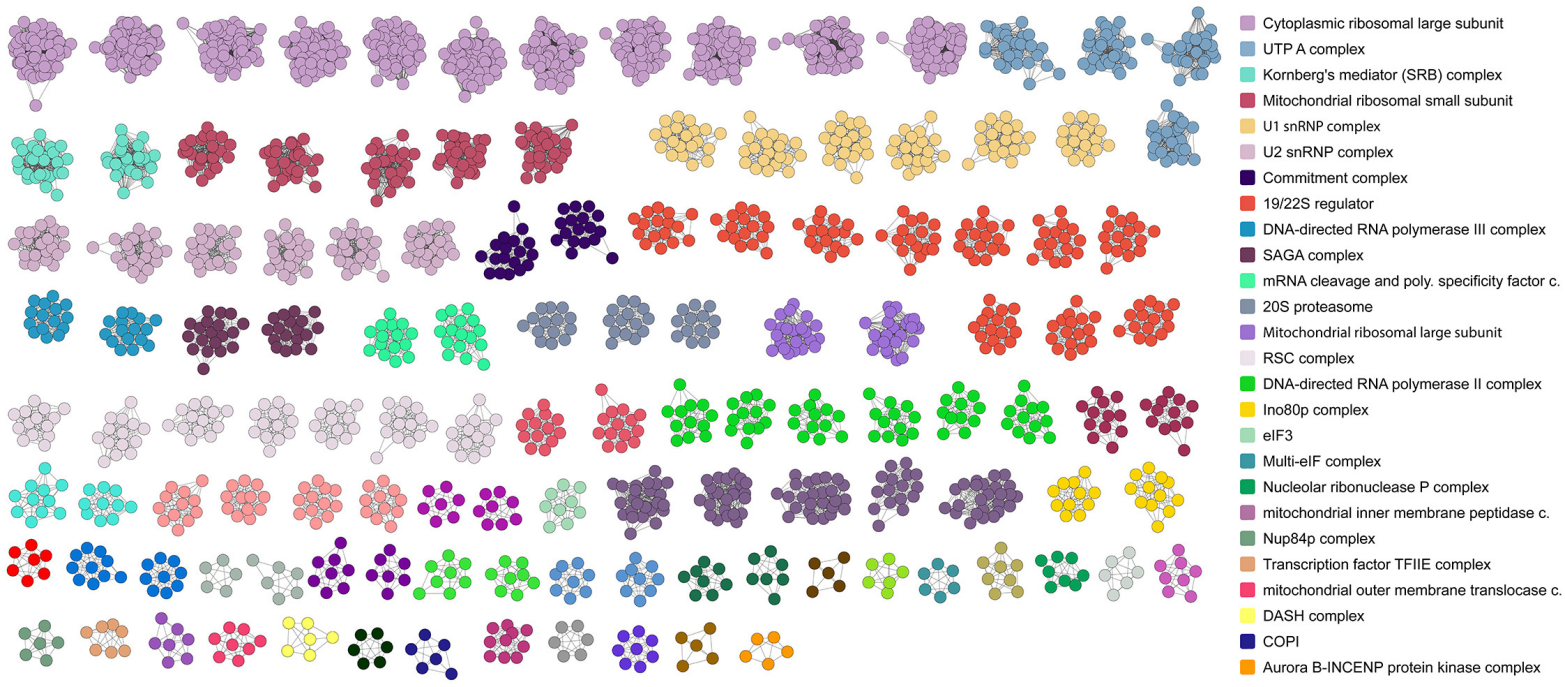
Budding yeast protein complexes predicted by SiComPre. Structure of many refined predicted complexes after dropping small abundance complexes. The colours are chosen according to the best matching reference complex. The legend shows some of the most well-known complexes (the full list can be found in S1 Table). Similarly coloured RCs match a single reference complex. These RCs can be considered different variants of the same complex.

**Fig 3:**
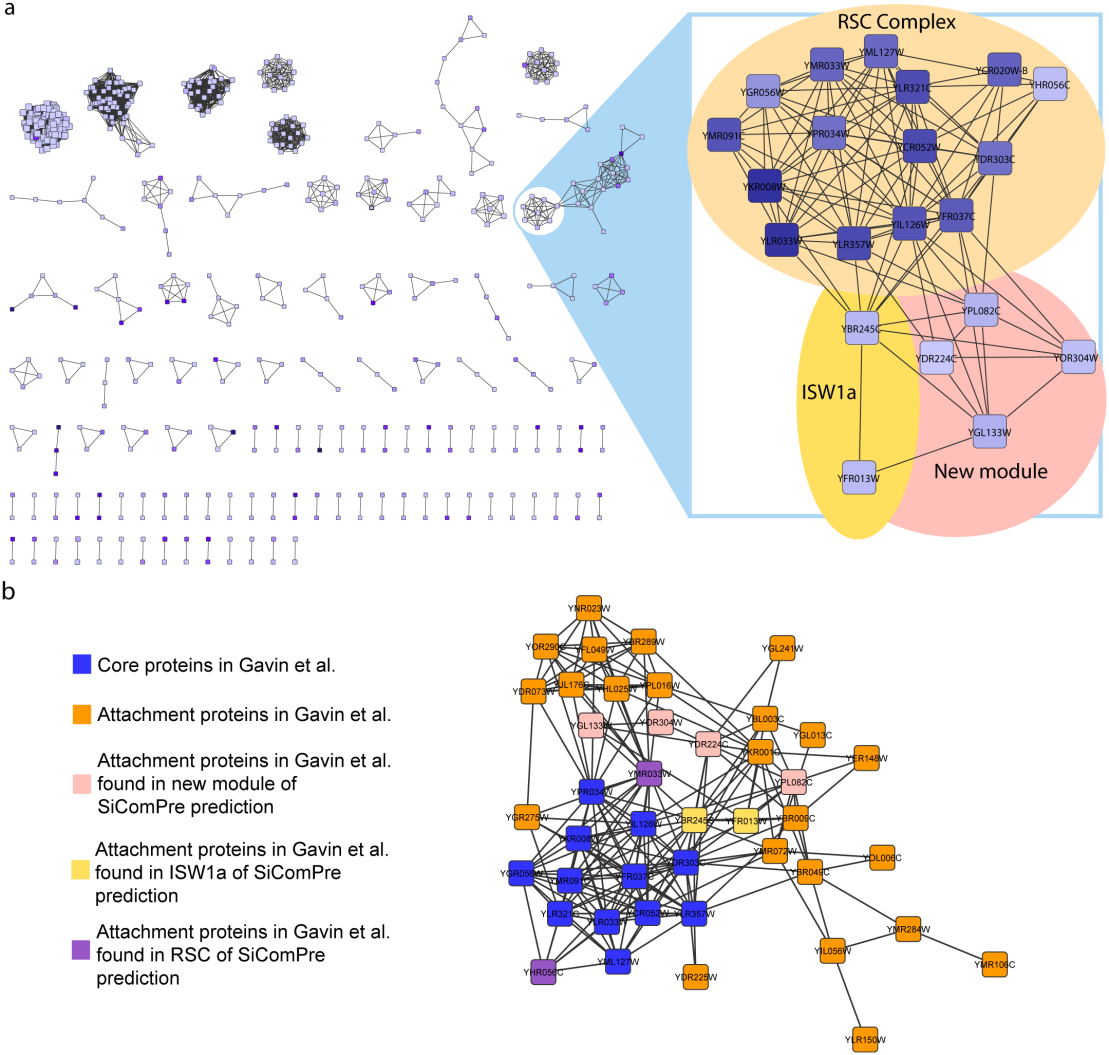
Relationship between predicted refined complexes (RCs). a, In the left part of this figure, nodes represent all the predicted RCs, edges represent overlap between RCs. Connected protein complexes share components above a threshold overlap (≥ 0.1). Node size corresponds to the number of proteins in the complex and the colour represents the quantitative prediction with darker colour meaning higher abundance. Some of these connected components match the same reference complex with every node representing a complex variant. In the right part of this figure, we merged all variant refined complexes that could be associated with the RSC complex, the colour depth of the nodes represent how many times a protein has been observed in a SC that match one of these RCs. In this case edges represent interactions between proteins found in the initial PPI dataset [23]. The three set of proteins with coloured background are named according to the corresponding reference complexes [9]. All the proteins of the reference RSC complex are found by SiComPre except YBL006C and YGR275W. These form a dense region with higher abundance corresponding to the core complex and a less dense auxiliary complex attached to it. Two of the proteins in the less dense region match the reference complex of ISW1a, suggesting a strong interaction between these two complexes. b, The core RSC complex and its attachments according to Gavin et al. [5] compared with the RSC complex predicted by SiComPre. Blue nodes are core proteins, while all the others are attachments according to Gavin et al [5]. Colour indicate whether they are predicted by SiComPre either to be in RSC complex (purple), ISW1a complex (yellow), new module (pink) or not bounded to RSC (orange). Edges represent interactions according to the initial PPI database [23].

Even after the clustering and optimization steps, we found that multiple RCs that differ in either size or exact structure (Fig 2) are associated to a single experimentally characterized complex. For instance several alternatives of the ribosomal large subunit were found, which could be different existing variants or be caused by the lack of rRNAs in the simulations. SiComPre also predicted several RCs that we could not associate with any characterized complexes. We identified some complexes containing up to six proteins and several of them showed high abundances (>1000 copies per cell). Two of these six-protein complexes (RC 222 and 272 in S1 Table) share four common proteins and, according to functional annotations, are related to nuclear transport processes. These and other predicted complexes in S1 Table call for further research on their possible existence and role in yeast cells. Similar overlaps between RCs are plotted in Fig 3. This graph represents all the RCs (nodes) and the protein content overlapping between them (edges). Larger protein complexes are associated with more alternative RCs by SiComPre. The alterative RCs of an existing protein complex could be merged to increase the precision of predictions. However, alternative RCs could also correspond to existing variation of the same complex and could thus lead to the discovery of other proteins that associate and have functional relevance in an already known complex. An example of possible auxiliary subunits of the chromatin remodelling RSC complex [34] is highlighted in Fig 3a. SiComPre predicted that the RSC complex often interact with the ISW1a complex [35] and a new module of four proteins (Fig 3a). A similar subdivision of protein complexes has been proposed by Gavin et al. [5], where proteins are either part of the core of a complex or are attachments or part of modules bound to the core proteins. The core group is preserved in most of the isoforms of the complex, while attachments and modules may give a different function to the complex. Their analysis is based on genome-wide mass spectrometry data thus can be directly used to validate SiComPre results. All but two of the proteins in the most abundant SiComPre predicted RSC complex are part of the core of the RSC complex of Gavin et al [5] and ISW1a and the four proteins in the new module of the SiComPre complex are all attachments of the Gavin RSC complex (Fig 3b). This shows that SiComPre can be used not only to quantitatively predict protein complex abundances but also to predict possible alternative compositions of these complexes.

As a control, we investigated whether protein complex abundances can be predicted simply by averaging the abundance of all the constituting subunits. We found that on average there is a 14-fold difference between SiComPre quantitative predictions of protein complexes and the average abundance of their constitutive subunits with low correlation between them (Pearson 0.159, Spearman’s 0.006 (S1 Text)). The importance of the use of actual protein abundances in predicting protein complex abundances can be also seen on the predictions of the few examples with literature data (Table 2).

### Prediction of human protein complexes

We also tested SiComPre on the human PPI [10] with protein abundances from a human osteosarcoma cell line (U2OS) [3]. We validated the results against the CORUM dataset of mammalian protein complexes [11] from which redundancies and complexes smaller than three proteins have been removed [10, 11]. This resulted in a new dataset of 324 non redundant human protein complexes, 39 of which were identified by SiComPre. Despite this relatively low match, our predictions outperform any other existing methods [10] (Fig 1B), predicting four more complexes than ClusterOne (Fig 1C). The low number of matched complexes is due to the lack of comprehensive experimental data, which cannot be compensated for by any prediction methods. It is possible that multiple instances of a predicted complex correspond to an existing, but so far unknown, complex. For instance, the high abundance six-protein complexes RC 145 and 504 (S2 Table) share five common proteins, which are all associated with snRNA binding (without our addition of any fictitious domain) and thus suggests the existence of these complexes. Indeed this complex appears under the name of LSM-complex in the extended CORUM dataset of 1685 characterized complexes from which SiComPre matches 295 protein complexes. Similarly, Complex RC 259 cannot be associated to any of the CORUM complexes (S2 Table) but it matches the pyruvate dehydrogenase complex [36] based on Uniprot protein descriptions. Examples of complexes which cannot be associated to any characterized complex are the five-protein complexes RC 365 and RC 391. These share four components and are associated with Rab related GTPase activity, vesicle formation and transport [37]. A few of the constituting proteins participate in the RCP-RAB11 and Rab geranylgeranyltransferase complexes but the whole complex does not show significant overlap with either of these. It is important to note that interactions between the constituting proteins of these complexes are always supported by known DDI, thus no fictitious domain had been added to predict these complexes.

It is hard to find validation for the predicted abundances of protein complexes in the considered osteosarcoma cell line (U2OS). SiComPre predicted 1.3 × 10^6^ ribosomes, which is in the same order of the number of ribosome identified in HeLa cells (3.3 × 10^6^) [38].

### Robustness of SiComPre predictions

SiComPre predicted 4456 different types of budding yeast protein complexes after two stochastic simulations and considering two separate time points. This is a surprisingly low number considering all the possible complexes that could appear from the initial PPI sub-network of interacting proteins of the proteome-wide yeast network (1622 nodes and 9074 edges). 2983 complexes were predicted by the first simulation with random initial position of each protein (we considered two time points of that single run). The second stochastic simulation with new, random initial settings, shared 1462 complexes with the first run. Similarity increases to almost 2030 complexes (68% of total hit counts) when complex with similar counterpart (overlap > = 0.75) between two simulations are considered. Moreover, only one simulation is enough to identify 90% of reference complexes, while the addition of a second simulation increases the percentage of predicted reference complexes by only 1% (S1 Fig). Addition of further simulations does not increase predictive capabilities (Fig 1). This suggests that most complexes robustly form independently of the stochastic noise in the initial layout of proteins in the various sub-volumes. Quite often the protein complex abundances also show extraordinary robustness. A good example is the methionyl glutamyl tRNA synthetase complex [39] with abundances 221^2^ and 225^2^ (actual simulated values to the square to predict real biological abundances) in two simulation runs with a perfectly predicted structure match (matching score = 1). Many other complexes also have small abundance variations between the two simulations (S1 Table) and only small fraction of yeast complexes show high sensitivity to noise in initial settings. For instance, the abundance of the mRNA cleavage factor complex (CFI) varied between 17^2^ and 30^2^ copies and the Pho85p/Pcl8p complex was not observed in the first simulation but 64 (8 simulated) complexes appeared in the second run. To get a broader picture for each complex we calculated the coefficient of variation (CV = standard deviation / mean) of its quantitative predictions. Only 20% of complexes show a CV > 0.5) in the case of the human protein complex predictions after three runs (S3 Table). In yeast, where only two runs were performed, 36% of complexes have CV > 0.5. Finally we compared the quantitative predictions resulted from two separate simulations and observed that quantitative predictions are also robust as the two simulations on the yeast data gave quantitative protein complex predictions with a Pearson’s correlation of 0.997, while based on results from the human data this correlation was 0.998 (S2 and S3 Tables).

### Effect of a drug on the human complexome

Tissue-specific protein data is emerging [40] and shows that protein expression and abundance can greatly vary between tissue [17]. SiComPre can take such tissue-specific information into account and thus give tissue-specific protein complex predictions, which could soon be useful in extending our knowledge of human protein complexes. Furthermore the tissue specific variations in protein levels in cancer and other diseases [41] could be translated into qualitative and quantitative predictions on protein complexes by SiComPre. These results could be used to associate complex abundances and compositions with diseases as novel therapeutic targets [20]. For instance the administration of a drug can influence the abundance of complexes or allow the formation of new complexes. As a proof of concept, we performed simulations on the human SiComPre model with the addition of Bortezomib, a proteasome inhibitor [42] (details in S1 Text), which is a highly characterized drug with known to affect the formation of protein complexes. Drug—protein interactions were collected from the Stitch database [43] and after performing a domain enrichment of interacting proteins we estimated the domains bound by the drug (S1 Text). We set the abundance of Bortezomib to 5000^2^ molecules, which is roughly the abundance of the most abundant protein we consider for human cells. We mapped protein complexes predicted with and without Bortezomib by finding the best matching complexes in the normal case to the complexes found after Bortezomib addition. Abundances of matching protein complexes were analysed by a t-test to find complexes which were perturbed in their abundance by Bortezomib (p-value <0.05). Protein complexes without a best matching complex were considered qualitatively altered by Bortezomib. We observed that the abundance of the Proteasome, the Anaphase-Promoting Complex, Prefoldin and the Multisynthetase complex were greatly perturbed by Bortezomib. We also observed that the composition of the above discussed snRNA binding LSM complex and several other predicted complexes were modified by drug treatment (S3 Table). Several of the altered complexes are involved in transcriptional regulation (constitutive proteins are known transcription factors [44]). We searched the literature for validation of the involvement of SiComPre predicted transcriptional complexes in Bortezomib treatment and found numerous transcription factors that could be implicated (S4 Table). These predictions cannot be trivially inferred from the direct interactions of Bortezomib [43] as most of the candidate transcription factors are part of larger complexes that are perturbed by Bortezomib. Thus, we can conclude that SiComPre could be used to predict qualitative and quantitative changes to complexomes upon drug treatment.

### Conserved protein complexes

**Fig 4:**
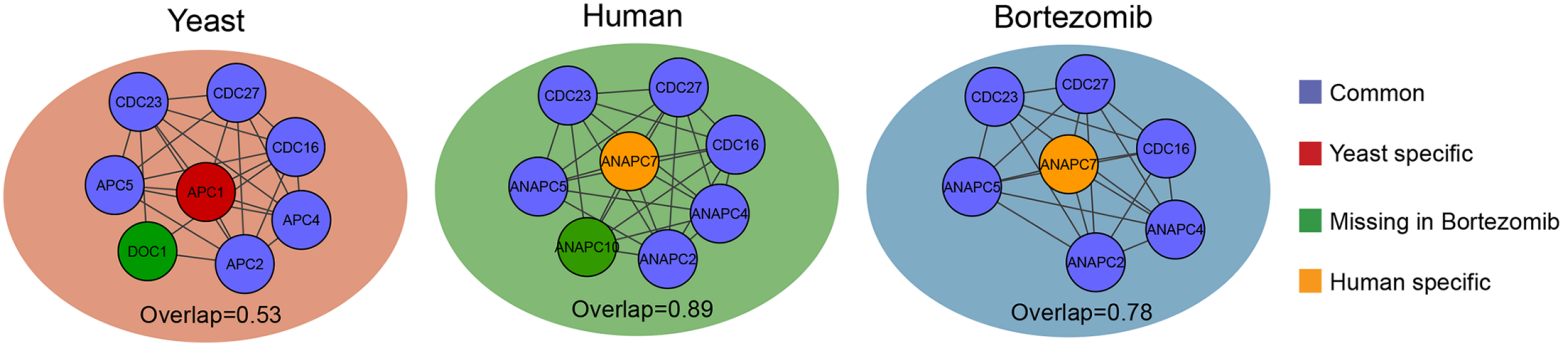
Variations in SiComPre predicted anaphase promoting complexes. The predicted structures of the APC complex in yeast, human and human after Bortezomib treatment. The reported overlap scores were calculated by comparing to the reference protein complexes discussed above. The lower score observed for the yeast is due to the larger APC complex size found in yeast [9].

Several protein complexes perform essential biological functions slowing down their evolution and allowing only co-evolution of their components [45]. We investigated how protein complex compositions and abundances change between organisms with the example of the Anaphase Promoting Complex (APC). Fig 4 shows the structures of SiComPre predicted APC in yeast, human and in human after Bortezomib treatment. All three SiComPre complexes show high overlap with the experimentally identified complexes [46]. The SiComPre predicted yeast APC complex shows an overlap of 0.68 with the core of the APC complex found by Gavin et al [5] and with one protein exception, fully matches the orthologs of the predicted human APC (Fig 4). The Bortezomib treatment seems to cause a loss of ANAPC10 (the yeast ortholog is Doc1) from some of the SiComPre simulated APC complexes (Fig 4). Such loss of ANAPC10 could cause an S-phase block in the cell cycle [47]. The constituting proteins are not the only variations revealed by SiComPre. As expected, SiComPre predicted the abundance of APC in human almost one order of magnitude higher than in yeast. Unexpectedly SiComPre also predicts that the addition of Bortezomib further doubles the abundance of APC in human (S2 and S3 Tables), although the majority of these complexes might be defective due to the lack of ANPC10.

### Quantification of limiting subunits

**Fig 5:**
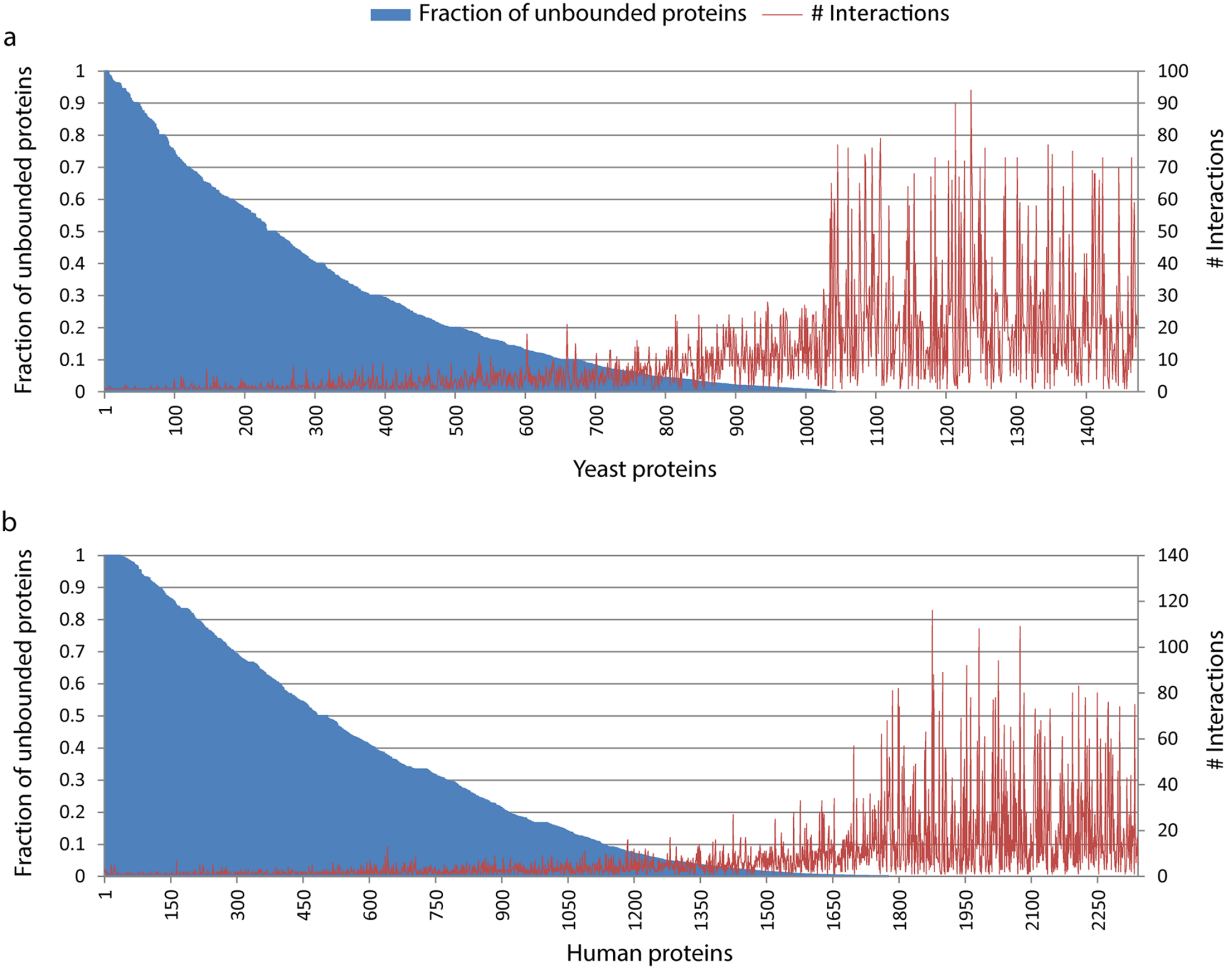
Fraction of unbound subunits. We calculated the predicted number of unbound proteins in a cell by subtracting the number of protein complexes from total number for each protein. a, Fraction of free proteins in yeast plotted together with the number of their interactions based on the Collins PPI network [23]. b, fraction of free proteins in human and the number of their interactions in the considered PPI dataset [10].

Thanks to simulated complexes it was possible to estimate the number of unbound proteins. This can help us to identify which proteins are fully bound in complexes, thus might limit the formation of other complexes. As expected there is a negative correlation between fraction of unbound proteins and the number of interactions of proteins (Pearson’s correlation -0.43 for yeast and -0.39 for human data) as with more possible interactors there is a higher chance of ending up in a complex. Interestingly there is no correlation between the fraction of free subunits and the abundances (Pearson’s correlation 0.03). A high number of proteins are present in high abundance have a few interactors, but fully used up in complexes (Fig 5 and S5 Table). For instance, in yeast, TEF4 (YKL081W) has only 6 interactions, present in 102,000 copies, which are all bounded in complexes. This and several others with low (many cases 0) free abundance could be limiting factors in protein complexes.

## Discussion

Here we introduced a simulation based protein complex prediction method (SiComPre) that outperforms existing tools in qualitatively predicting the components of protein complexes and provides for the first time quantitative predictions on protein complex abundances. SiComPre incorporates multi-source information and our results show that the addition of domain information and protein abundances both increase the qualitative prediction of protein complexes.

Membrane-bound protein complexes are often difficult to be detected. Identification of membrane protein interactions [48] will allow SiComPre to predict complexes in the membrane with higher precision. In the future SiComPre could also identify variations in the complexome of different organisms and in different tissues of the same organism [40, 49] as tissue-specific data becomes available. SiComPre will provide increasingly reliable predictions with the growing availability of human proteins data such as cellular sub-localization, abundance and binding/unbinding rates. This will enable the discovery of new human protein complexes and the understanding of the relationship between their variations and complex phenotypes. Another possible expansion comes from a recent tool that can predict protein abundance changes through the cell cycle [50]. This data could be used as input for our simulations allowing SiComPre to predict the dynamic of the complexome throughout the cell cycle. In summary, SiComPre opens a new area of computational analysis of the complexome.

### Software availability

All SiComPre applications and datasets are provided to the community on a dedicated website (www.cosbi.eu/research/prototypes/sicompre). The scripts are available as supplementary S1 File.

## Supporting Information

S1 Table SiComPre predicted budding yeast protein complexes together with their predicted abundances in Microsoft Excel format. (XLSX)

S2 Table SiComPre predicted human protein complexes together with their predicted abundances in Microsoft Excel format. (XLSX)

S3 Table SiComPre predictions on the effect of bortezomib on human protein complexes in Microsoft Excel format. (XLSX)

S4 Table Transcription factors bounded to altered complexes in the simulations with Bortezomib added. This table is in Microsoft Word format.
(DOCX)

S5 Table Predictions of the fraction of unbound proteins by SiComPre simulations of the yeast and human data in Microsoft Excel format. (XLSX)

S1 File Zipped scripts to run SiComPre. (RAR)

S1 Text Supplementary materials with text. (PDF)

S1 Fig Effect of the number of considered simulations on qualitative predictions. (DOCX)

S2 Fig Composite scores of protein complex qualitative predictions after two simulations with actual (left) and average (right) protein abundances. (DOCX)
