# Supplementary material for: Qualitative and Quantitative Protein Complex Prediction Through Proteome-Wide Simulations
Source: PLoS Comput Biol. 2015 Oct 22;11(10):e1004424. doi: 10.1371/journal.pcbi.1004424 (PMC4619657; doi:10.1371/journal.pcbi.1004424)
Supplement: S4 Table — This table is in Microsoft Word format. (DOCX) [file pcbi.1004424.s004.docx]

**S4 Table. Transcription factors bounded to altered complexes in the simulations with Bortezomib added.**

This table lists all the transcription factors identified in altered complexes and for each of them explains their function and documented interactions with Bortezomib, together with references.

| **Altered Transcription Factors** | **Function of encoded genes** | **Interaction with Bortezomib** | **References** |
| --- | --- | --- | --- |
| **Vitronectin** | Cell adhesion and spreading factor | Metastatic cancer is strongly related to cell adhesion. Therefore an alteration of these genes may have an big impact on cancer progression | [[34](#_ENREF_34)] |
| **CREB1** | Phosphorylation-dependent transcription factor that stimulates transcription upon binding to the DNA cAMP response element (CRE) | Another transcription factor modulated by Bortezomib in our model was activating transcription factor 3 (ATF3), a member of the mammalian activation transcription factor/cAMP responsive element-binding (CREB) protein family of transcription factors and an immediate early response gene induced in cells exposed to a variety of stress stimuli, including ER stress and proteasome inhibition. | [[35](#_ENREF_35)] |
| **Upstream-binding protein 1** | Involved in regulation of the alpha-globin gene in erythroid cells | Unknown | [[36](#_ENREF_36)] |
| **Cell division cycle 5-like** | DNA-binding protein involved in cell cycle control. May act as a transcription activator | Unknown |  |
| **Zinc finger protein 454** | Unknown | The proteasome inhibitor Bortezomib is a potent inducer of zinc finger AN1-type domain 2a gene expression. Although it is not yet known which genes are encoded by this transcription factor, it belong to the same domain family of ZNF AN1-type domain 2a, therefore there might be an interaction. | [[37](#_ENREF_37)] |
| **Nuclear factor 1 A-type** | Binds the sequence 5'-TTGGCNNNNNGCCAA-3' present in viral and cellular promoters and in the origin of replication of adenovirus type 2 | Unknown |  |
| **Nuclease-sensitive element-binding protein 1** |  | No direct connection between bortezomib. However, it has been found downregulated in Bortezomib reistant clones | [[38](#_ENREF_38)] |
| **Activity-dependent neuroprotector homeobox** | May mediate some of the neuroprotective peptide VIP-associated effects involving normal growth and cancer proliferation. | Unknown |  |
| **Zinc finger and BTB domain-containing protein 43** |  | See Zinc finger protein 454 |  |
| **Steroid hormone receptor ERR1** | Important regulator of MCAD promoter. Binds to the C1 region of the lactoferrin gene promoter. Induces the expression of PERM1 in the skeletal muscle. | PERM1 is an estrogen receptor and ER is altered after Bortezomib treatments. | [[39](#_ENREF_39)] |
| **Zinc finger protein 606** |  | See Zinc finger protein 454 |  |
