# Supplementary material for: Qualitative and Quantitative Protein Complex Prediction Through Proteome-Wide Simulations
Source: PLoS Comput Biol. 2015 Oct 22;11(10):e1004424. doi: 10.1371/journal.pcbi.1004424 (PMC4619657; doi:10.1371/journal.pcbi.1004424)
Supplement: S1 Text — (PDF) [file pcbi.1004424.s007.pdf]

Supplementary Materials (S1Text) to:

Qualitative and quantitative protein complex prediction through proteome-wide simulations

Simone Rizzetto[1], Corrado Priami[1,2,*], Attila Csikász-Nagy[3,4,*]

[1] The Microsoft Research-University of Trento Centre for Computational Systems Biology, Piazza Manifattura 1, Rovereto, 38068, Italy

[2] Department of Mathematics, University of Trento, Povo (TN) 38100, Italy

[3] Department of Computational Biology, Research and Innovation Centre, Fondazione Edmund Mach, San Michele all'Adige 38010, Italy

[4] Randall Division of Cell and Molecular Biophysics and Institute for Mathematical and Molecular Biomedicine, King's College London, London SE1 1UL, United Kingdom

Contents

I Supplementary text 2

1. Model generation 2
2. Simulator settings 2
3. Clustering 4
4. Qualitative prediction 4
5. Quantitative prediction 7
6. Use of alternative data sources 8
7. Application of SiComPre to drug discovery 8
8. Future perspectives 9
9. Documentation to SiComPre scripts and requirements 10

II. Supplementary Figures 12

III. Supplementary Tables Description 19

IV. Tables 22

V. References 23

I Supplementary text

1. Model generation

In the SiComPre framework proteins interact with each other through specific regions called binding sites. The modelling structure comes from process calculi [1], frequently used to model interactions of entities in concurrent systems. We consider proteins as the main entities of the system and domains as the interface through which proteins communicate. Therefore, an established communication indicate an interaction between two entities, moreover the communication channel allow changes in the state of the entity (which is not considered here). This is the basic idea of BlenX [2], a stochastic simulation framework based on process calculi and the Gillespie's stochastic simulation algorithm [3]. Instead of considering the cell as a well-mixed container of molecules we split the simulation space in 4096 sub-volumes (SV) and allow only local interaction of proteins and their diffusion to neighboring compartments in the imaginary 2D simulation space (see details below). We chose protein binding sites according to protein domains using SMART [4] and check if there exists a known interaction between the identified domains of proteins involved in a protein-protein interaction (PPI). Unfortunately this is not always the case. To consider the remaining PPIs in our analysis, we tried various strategies:

Full: If a DDI is not found between partners of a PPI then specific fictitious interacting domains are added to these proteins.
noFictitious: A PPI is considered only if a corresponding DDI is found. This strategy yields less false positives, but the false negatives increase.
Function: We add fictitious domains only if the proteins of the binary interaction are involved in the same biological function according to the MIPS database [5].
As mentioned in the main text, the use of MIPS functions led to the highest composite scores (**Figure 1**) and this *Function* strategy enabled us to consider 7618 protein-protein interactions from the original Collins et al PPI dataset of 9074.

Binding and unbinding rates for all molecules are set to 100 and 1 arbitrary unit respectively, thus favoring complex formation, but allowing unbinding of proteins that might be present in low abundance. Therefore, only protein abundances but not the specific binding rates have an influence on the propensity of possible reactions, with higher abundance proteins having a higher chance to participate in a binding reaction. Clearly this is a point that could be easily updated with specific binding and unbinding rates of each molecule in case such data would be available.

2. Simulator settings

A brief description of the algorithm is showed in the online methods section, here we explain how we handle the simulation space enabling us to parallellize the computation.

In most Gillespie-type exact stochastic simulations[6], the simulator calculates the propensities for each possible reactions. However this is not always necessary as during each Gillespie step we do not need to recompute the whole set of interactions, only those that have been modified during the last performed reactions. In classic Gillespie algorithm space is not explicity assumed and the diffusion of molecules is considered as part of the reaction rates. To simulate protein complex formation it is absolutely important to consider space as well, because closely located proteins, or

proteins that already participate in the same complex should have higher probability to bind each other compared to those that are far. Therefore, simulation algorithms that don't consider space like Gillespie or ODE modeling cannot capture the right behaviour in complexation and decomplexation of proteins, leading to the formation of long filament-like structures.

If we would consider a single simulation space then real large complexes formed of a few types of high abundance proteins could be formed as all possible protein-protein interactions between protein complexes could be allowed. The problem is that three proteins of complex that all bind to each other might not form the proper triangular structure of binding each other, rather they bind to other proteins available in the solution. For instance, a triangle formed by protein A, B and C where A interacts with B, B interacts with C and C interacts with A. This might not be observed because protein C in the temporary filament A-B-C have a chance of 1 over the abundance of A to bind with exactly the A protein already in the complex. This will generate filaments like A-B-C-A-B-C-A. When space is considered the amount of A proteins within one SV is very limited, thus C will bind to A closing the triangle. Similar problems could occur with larger protein complex, but the use of small sub-volumes reduces the chances of unrealistic complex formation. As explained in the main text, we consider the square root of the actual protein abundances. This further helps us to reduce the chances of such chain forming reactions. Furthermore the use of square root of abundances reduces the computational needs by greatly reducing high abundance protein levels while only minimally changing the levels of low abundance proteins.

To deal with this, we consider a two dimensional discretized simulation space and diffusion of molecules between neighboring compartments. A two dimensional 64×64 square lattice with 4096 compartments is enough to reduce the possibility of protein complex aggregation to a level that it is not interfering with normal protein complex formation. The proper 3D structure of the cell and known localization of the proteins could be used in a future version to make the simulation space biologically realistic, at this stage we just focused on reducing global mixing of proteins to a tractable level.

Diffusion is also considered as a reaction when molecules move from one SV to a neighboring one, but if in one Gillespie step no diffusion or reaction transitions occured we do not need to recalculate propensities for the given SV. Due to the high number of propensities to be computed we need high computational power, but the independency of distant SVs enables us to use massively parallel architectures like GPUs. Indeed this is problem that can have a considerably advantage from GPU computing[7]. To implement our algorithm we used CUDA, a GPU computing platform provided by NVIDA. Unlike CPUs, GPUs have a parallel architecture that emphasizes executing many parallel threads slowly, rather than executing a single thread very quickly. CUDA provide to developers a set of functions to develop concurrent algorithm that match parallel architecture of a GPU.

Proteins diffuse randomly at discrete time steps according to the Flick's law [8]. The diffusion time for each molecule is calculated according to its diffusion rate and corresponds to the time necessary for all proteins to diffuse in the neighbor SV:

$$\tau_i = \frac{l^2}{2 * dim * D_i} \quad i = 1, \dots, M$$

where l is the lattice size, dim is the number of dimensions, D is the diffusion rate and M is the number of protein types. We chose $l = 0.1, dim = 2$ and $D_i = 1$ for every i. The values obtained give the timestep in which a diffusion reaction (move to neighbor) occurs. Proteins of type i diffuse at time $\tau_i * n$, where n is an integer incremented at every diffusion of proteins with type i. A random number is generated for each protein that has to be diffused and it will decide in which direction the protein is moving. In case a protein is bounded to another one, both proteins have to reach their diffusion time before moving to the same lattice. To limit the amount of proteins in a SV, the probability of moving to a neighboring lattice is proportional to the number of proteins in that lattice. The probability of a complex c to move in a sub-volume s is

$$P = \frac{MC - (A_s + S_c)}{MC}$$

where MC is the maximum protien number per SV (200 for yeast simulations and 400 for human simulations), A_s is the current number of proteins in sub-volume s, S_c is the size of the complex c. After a diffusion step the simulation time is updated to the diffusion time. In the interval between diffusion times an optimized instance of Gillespie Direct Method (DM) runs in each lattice allowing proteins to bind/unbind [3,8]. Compared to previous ideas [8], the reactants of our simulator are the binding sites, instead of the proteins.

3. Clustering

Our simulations return a list of complexes with high redundancies. We call these complexes *simulated complexes* (SC). Many of these SCs differ only in a few proteins and/or complexes could be connected through one or a few shared components (e.g. RSC bound to ISW1a as on Fig. 3 in the main text), thus we need both to split aggregated complexes and to merge complexes with almost equal constituents. We apply a clustering algorithm to the frequency matrix that represents how many times two proteins appear together in SCs. At this purpose we developed a weighted version of the IPCA algorithm. The original IPCA [9] has a weighting process for PPIs that count the number of shared neighbors of the two proteins involved in the interaction. In our version instead of counting shared neighbors, we sum the interaction score of each protein pairs from simulated complexes. We have found that the weighted IPCA algorithm gets higher *composite score* and *f-score* results when the SiComPre simulation based frequency matrix is used ad weighting input compared to the originally used Collins et al. PPI dataset[10]. The clustering will return a new list of complexes we call *refined complexes* (RC).

4. Qualitative prediction

4.1 Measures on prediction quality

To evaluate SiComPre we used various established measures of protein complex prediction performance:.

Overlapping [11]**:** using this value, one can overlap a predicted complex with one from the reference dataset.

$$Overlap(A, B) = \frac{|VA \cap VB|^2}{|VA| \cdot |VB|}$$

where VA is the set of proteins in complex A, analogous for VB.

Recall [11]**:** it corresponds to the fraction of complexes in a reference dataset that were correctly predicted, where P is the set of predicted complexes and B is the set of reference complexes (false negatives decreases this).

$$Recall = \frac{|\{b|b \in B, \exists p \in P, Overlap(p,b) > \omega\}|}{|B|}$$

ω is a threshold value. In all of our analysis we select a threshold value of 0.25, therefore we consider a match only if we have an overlap score greater than 0.25. This value was used in the literature to test all earlier methods [12] and has been suggested as optimal value by Bader et al.[11].

Precision [11]: it is the fraction of predicted complexes that find a matching complex in the reference dataset (false positives decreases this).

$$Precision = \frac{|\{p|p \in P, \exists b \in B, NA(p,b) > \omega\}|}{|P|}$$

F-score [11]: the harmonic mean between precision and recall.

$$F - score = \frac{2 \cdot Precision \cdot Recall}{Precision + Recall}$$

Sensitivity [13]: the fraction of proteins of complex i which are found in a predicted complex j, $Sn_{i,j} = T_{i,j}/N_i$, $T_{i,j} = Vi \cap Vj$ and $N_i = |Vi|$. While, *the reference complex-wise sensitivity* is the maximal fraction of proteins of complex i by its best-matching predicted complex $n_{co_i} = max_{j=1}^{m} Sn_{i,j}$. Finally the general sensitivity or *predicted complex-wise sensitivity* is the weighted average of *real complex-wise sensitivity* over all complexes

$$Sn = \frac{\sum_{i=1}^{n} N_i Sn_{co_i}}{\sum_{i=1}^{n} N_i}$$

Positive Predictive Value [13]*:* the number of proteins in predicted complex j which belong to a reference complex i over the total number of proteins of predicted complex j assigned to all complexes, $PPV_{i,j} = T_{i,j}/\sum_{i=1}^{n} T_{i,j}$. As above there is also a predicted complex-wise predictive value, $PPV_{cl_j} = max_{i=1}^{n} PPV_{i,j}$. While the general PPV is

$$PPV = \frac{\sum_{j=1}^{m} T_{.j} PPV_{cl_j}}{\sum_{j=1}^{m} T_{.j}}$$

where $T_{.j} = \sum_{i=1}^{n} T_{i,j}$.

Accuracy [13]: geometric accuracy is the geometrical mean of Sn and PPV, $Accuracy = \sqrt{Sn \cdot PPV}$

Maximum Matching Ratio [12]: is a new measure proposed for complex prediction evaluation in the original publication of ClusterOne [12]. MMR finds the better correspondence between predicted

complexes and reference complexes and can be solved as a maximum bipartite matching in weighted graph, where nodes corresponding to a predicted complex are connected with an edge to a node representing a reference complex and its weight is the overlap between these complexes.

Composite score [12]*:* it is the sum of *accuracy*, *MMR* and *recall* with a recall threshold strictly greater than 0.25. The same recall threshold has been used for f-score calculations as well.

4.2 Qualitative results

We evaluate our qualitative predictions by considering different criteria, datasets and organisms. As a principal organism for evaluation we used data on the well characterized model organism *S.cerevisiae*. When comparing SiComPre with earlier methods we chose parameter value according to the values reported in the ClusterOne prediction evaluation analysis [12]. We further tested a few newer methods that were not originally considered in the ClusterOne analysis [12]. For these methods (IPCA and PEWCC) we selected parameters according the original publications [9,14]. When reference datasets contained complexes with two components we used parameter values optimized for this in the ClusterOne paper. We tried to further optimize the parameters of these other methods, but could not find parameters that would provide better composite scores than the default values could give. We also tested SiComPre against data on *Homo sapiens*. In this case literature offers less complete datasets, hence this evaluation cannot be considered reliable as the Yeast one. For Yeast we used dataset of complexes retrieved from manually curated complexes, MIPS, SGD and CYC08, while for human we used the dataset of non-redundant human protein complexes [15]. It has been created from the list of human protein complexes in CORUM with similar complexes merged in case they have a Simpson's coefficient greater than 0.5.

Simpson's coefficient is defined as a similarity between two complexes:

$$Similarity(A,B) = \frac{|VA \cap VB|}{\min(|VA|,|VB|)}$$

Where A and B are two complexes, while VA and VB represents the set of proteins in complex A and B respectively.

Additionally, protein complexes with less than 3 proteins have been removed. To compare the performance of different methods we filtered out complexes which proteins are not covered by the initial PPI dataset, after this steps we removed all the complexes of size lower than 2. In **Figure S2a** we show that the *composite scores* of SiComPre outperform all other method, and also *f-scores* of multiple versions of SiComPre are better than any other methods could reach (**Figure S3a**). It could happen that a predicted complex match more than one reference complex with an overlap greater than 0.25 (default threshold established in the literature) which might lead to a biased *recall*. For instance, a ClusterOne predicted complex (consisting of YDL047W, YFR040W, YJL098W, YGR161C, YOR267C and YKR028W) matches three CYC08 reference complexes (Sap190p/Sit4p complex, Sap155p/Sit4p complex and Sap185p/Sit4p complex). Similarly, SiComPre predicted RC 687 matches three complexes (transcription factor TFIID complex, SAGA complex and SLIK (SAGA-like) complex) with this 0.25 overlap threshold. In order to test the effect of this bias we calculated how the *composite score* and *recall* change considering only the best matching complex

for every predicted complex (**Figure 2b**), and also plotted the *f-score* measures according to this *updated recall* (**Figure 3b**). SiComPre outperforms other methods even in these tests. Finally, **Figure 4** depicts the composite-score for complexes predicted for human. In this case only IPCA is slightly better than SiComPre, but it has a lower f-score (**Figure 5**). We also tested the composite score of the set of simulated complexes against many other methods (**Figure 6**), this time we removed all the complexes of size lower than 3.

5. Quantitative prediction

To predict the quantity of each refined complex for each simulated complex we identified the refined complex for which it has the highest overlap score [12]. This way, we were sure to consider each simulated complex only once. The predicted abundance of each refined complex is the square of the number of simulated complexes matching it, since we were considering the square root of protein abundances. It is possible to improve the prediction measured on the budding yeast datasets by the *composite score* by removing complexes of size greater than 16 with abundance lower than 6 or the alternative *f-score* [11] could be optimized by removing complexes with size smaller than 3 and abundance lower than 3. The same size and abundance threshold were used for SiComPre-LG and SiComPre-SM also in the human protein complex predictions.

To predict abundances of refined complexes (RCs) we summed the total amounts of all simulated complexes (SCs) that had the highest overlap with the given (RC). In this way we considered all the simulated complexes only once. Thus the predicted abundance of each refined complex is the square of the sum of matching simulated complexes. A summary of the predicted value can be found in supplementary material. To validate the quantitative prediction with literature data we first sum the abundance of RCs that match the same reference complex and then we calculated the square of this value. For an example we predict the presence of 110,889 copies (333 total simulated complexes associated to 3 RCs, see **Supplementary Table S1**) of yeast proteasomes. In the main text we also provided other example of our quantitative predictions. Next, we checked whether these information can be used to further refine our qualitative prediction. Initially, we removed complexes with low abundance, but we observed that both *f-score* and *composite score* were decreasing (not shown). Thus, we tried more complex filtering strategies: in the first strategy we removed complexes bigger than a given size and with abundance lower than a given threshold. In the second strategy we removed complexes smaller than a given size and with abundance lower than a given threshold. In supplementary material we show that the optimal values for these strategies are independent from which yeast protein complex dataset we used for testing our predictions (**Figure 7).** In all cases we found the same parameter range where *f-score* is maximal with small size complexes removed or *composite score* is maximal with large size complexes removed. This finding highlights that the two scoring system differentially evaluates the errors in predicting large and small size complexes. We also checked whether protein complex abundances can be predicted simply by averaging the abundance of the single subunits. For every refined complex RC, we calculated the average abundance of its constitutive subunits and compared these values with the square of the quantitative prediction and found that there is a 14-fold difference between these values. The Pearson and Spearman's correlations are 0.159 and 0.006 respectively. Finally, we tested if our predictions based on actual protein abundances can provide any improvement on the simple method, which would consider fixed protein abundance. For this test we considered the average of all protein abundance. SiComPre quantitative results show a higher

correlation with experimental results than the affixed protein abundance method (Pearson's correlation = 0.408, Spearman's correlation = 0.407, instead of 0.259 and 0.24 of the averaged-based method, although these correlations are not significant as they are based on only 8 measurements. As new quantitative data becomes available we might be able to show a more significant correlation between experimental and SiComPre predicted data.

6. Use of alternative data sources

In order to assess the reliability of protein complexes we verify whether each protein of a complex is involved in the same biological function. Yeast and Human protein-functions relations are retrieved from MIPS [16] and GO [17] respectively. In column "Functions" of the **Supplementary Table S1** and **S2** we list all the function terms and their p-value for a given complex according hypergeometric distribution. Complexes in which most of the proteins are involved in the same functions are more likely to be real complexes, however it could be the case that our binding strategy by the introduction of fictitious domains based on PPI, DDI and functional annotations lead to false positive predictions. For this reason we added a column that represents the fraction of fictitious domains involved in complexes. Analyzing this value we identified different scenarios. Big size complexes usually have a high fraction of fictitious domains, probably because all the proteins have similar functions. In these cases fictitious interactions make the protein complex denser but in most cases the same proteins could have been observed as a single (although less dense) complex even without the fictitious domains. A second possible scenario for the case of the ribosome is that ribosomal proteins do not bind directly rather they interact through rRNAs. Indeed both yeast and human ribosomes have a fraction of fictitious domains around 0.8. A more detailed analysis of the biological functions associated to constituents of properly predicted reference complexes but containing high fraction of fictitious domains shows that the most occurring GO and MIPS functions are related to DNA and RNA binding (e.g. histone binding or splicing). In the possible third scenario the set of proteins in a predicted complex have a high fraction of fictitious domains leading to false positive predictions, because it is more likely the predicted complex is a functional module rather than a real protein complex.

7. Application of SiComPre to drug discovery

Variation in the complexome leads to variation in phenotype. Therefore everything that affects the complexome, like drugs, can have a major impact on cellular behavior. We tested how a proteasome inhibitor drug, called Bortezomib affects the complexome in SiComPre simulations. To identify the protein-drug interactions we used the STITCH database [18], from where we considered interactions with a confidence score greater than 0.7. To select the right binding site/domain of bortezomib, we performed a domain enrichment using DAVID web-tool [19,20]. Next, for every protein-drug interaction we checked if the protein has one of the drug interacting domains identified in the last step and we add an interaction between the drug and that specific domain of the protein. If such domains are not present in a protein, then we add an interaction between the drug and the fictitious domains of the interacting proteins. This approach predicts that Bortezomib may bind with seven PFAM domains (PF00149, PF07992, PF00012, PF01851, PF00070, PF10584 and PF00227). Abundance of the drug was arbitrary set to 5000 in our simulation, which corresponds roughly to the highest protein abundance observed (5000^2). Clearly it could be interesting to test the dosage effect of the drug, with this current setting we were aiming to affect all targets of bortezomib. After

running the simulations with these settings the new complexes and their abundances were associated with complexes identified in the drug-free condition. We associated each of the “normal condition” protein complexes with its best matching complex after drug treatment while drug induced complexes that were not observed without the drug are listed in a separate sheet in **Supplementary Table S3**. We noticed bortezomib induced changes in protein complex abundances (based on a t-test on 3 runs with and without the drug) in Ribosome, Proteasome, Anaphase-Promoting-Complex, LSM complex, Prefoldin and Multisynthetase complex. After a more careful look we realized that in some of these cases the abundance of the sum of complexes associated with a reference complex does not change, rather the exact composition of the complex changes to another variant of the reference complex that appeared in low abundance in the drug-free case. For instance, in the case of the APC the protein ANAPC10 is missing from the complex after the drug treatment. Some of the altered complexes are involved in transcriptional regulation (constitutive proteins are known transcription factors). A list of human transcription factors can be retrieved from AnimalTFDB [21]. We checked the function of each transcription factors and validate their possible involvement in Bortezomib treatement against literature data in **Table 1**. This shows that SiComPre can be used to test how drugs effect the complexome in a qualitative and quantitative manner.

8. Future perspectives

SiComPre opens to way to a completely new computational analysis in the field of systems biology. The computationally predicted complexes could be used to identify new complexes associated to diseases in different ways. First, one can do an OMIM [22] enrichment to associate complexes to diseases. Indeed, it is already known that proteins of the same complex have a probability higher than random to be involved in the same disease [23]. Based on this data, the SiComPre predicted complexes associated with diseases could be used as novel therapeutical targets [24]. Furthermore, experiments revealed various proteomic levels in cancer and other diseases, but such variations are also observed between different tissue types [25,26]. Such specific protein abundance levels can be used as input of SiComPre to predict the complexes with perturbed composition or abundance in the given condition. These could lead to predictions of new biomarkers. Another possible expansion comes from a recent tool that can predict protein abundance changes throughout the cell cycle [27]. This data could be used as input for our simulations allowing SiComPre to predict the dynamic of the complexome throughout the cell cycle.

In this study we show that our qualitative and quantitative predictions are consistent with the current knowledge, but we can further improve SiComPre in various ways. Some SiComPre (and also by the use of other methods) predicted complexes are not consistent with the compartments the actual proteins have been identified. Therefore we filtered out all the complexes which contained proteins that were shown to localize in different compartments. Moreover many complexes are localized in membranes, hence could be bind to proteins in two organelles, in such case we expect to have at least one membrane protein in the complex. **Figure 8** shows how the composite score of different methods changes if we remove such questioned localization complexes. (For this analysis the list of membrane proteins were retrieved from a recent study on membrane protein interactions in yeast [28], while protein localization data came from the COMPARTMENTS database [29].) SiComPre greatly outperforms all other methods is this compartmentalization corrected case.

However, using compartments as initial constrains of the simulation might limit the degree of freedom of the system and by this reduce the noise of our results. Indeed, a realistic compartmentalization of the simulation space would allow proteins to stay in a restricted region. In this way many complexes that were not predicted so far, due to the small probability to observe an interaction between low abundance proteins, will have higher chance to form. As explained above SiComPre predicted complexes which show a high overlap with reference complexes but have a high fraction of fictitious domains are often associated with "non-protein binding" (e.g. rRNA binding, DNA binding, ATP binding) functional terms. Finally, SiComPre allows the use of proper binding/unbinding reaction rates and diffusion constants which are related to the strength of a bond between proteins. Large scale identification of such rates could be done by molecular dynamics simulations of binding between proteins with known structures. As such data becomes available it could be easily incorporated into the SiComPre workflow.

9. Documentation to SiComPre scripts and requirements

In order to execute the scripts of SiComPre (**File S1**) the user needs to connect to our database, where data from the used resources is collected. This can be downloaded at www.cosbi.eu/index.php/research/prototypes/sicompre. Alternatively they can build their initial data files, in case interested about other organisms or want to start with other initial data. In this case they should follow the structure found in this database. The minimum hardware requirements are: a GPU device supporting CUDA and 4 GB of RAM. The typical simulation time of the yeast proteome analysis on a machine with NVidia GTS 360M, 4GB RAM and Core i7 2.6 Ghz is 8 hours, while the human proteome requires about 1 day of computation. The software is built for Windows. Additionally, users who want to generate new models need to have Java and MySQL installed. Python is needed to run the optimization script and the script to generate the CSV files that are summarizing results.

A detailed explanation of the script parameters can be found in the readme file in the pipeline package. In the first step of the pipeline one can generate a new model starting from a dataset in the database according different binding strategies. For example:

java -jar GenModel.jar -s 2 -i Collins -f data/prot_collins.txt -o model_collins.xml

in this case we generate a model for yeast with Collins PPI and function binding strategy. Next we can run the simulation. This step could take some time, depending on the performance of the used computer.

SiComPre10.exe -f model_collins.xml

Once the simulation is over the list of complexes are stored in a file called *resultsComplex.out* and one can use the results script to measure the quality of the simulated complexes.

java -jar Results.jar

These scores refer to the whole reference dataset, instead for the published results we removed complexes that contain proteins that are not in the initial PPI (they have no validated protein interactions). This script returns also the list of complexes in a different format that can be used as an input for the further scripts.

In the next step on can build the frequency matrix with the complexes structure file retrieved from the simulation.

java -jar FrequencyMatrix.jar complex_structure.out

and one can cluster the resulting file with IPCAw to retrieve the list of refined complexes.

IPCAw.exe -Gint_network.txt -S2 -P2 -T0.5 -Oresults_quali.txt

SBML format does not allow '-' character in the definition of species and protein like 'YPL249C-A', these have been translated into 'YPL249CA', therefore one has to either replace 'CA ', 'WA ', 'CB ' and 'WB ' into 'C-A ', 'W-A ', 'C-B ' and 'W-B ' or remove '-' from the reference dataset
One should repeat the steps from the simulations till this point to have two results. Finally one can generate a table like **Supplementary Table 1 and 2**.

python generateCSV.py results_quali.txt results_C1_1.txt results_C1_2.txt int_network_2.txt fictitious_interactions.txt funcat.txt > prediction.csv

It is also possible to filter out complexes according to one of the strategies to optimize the *f-score* or the *composite score* by removing small or large complexes with low abundance.

python optimize.py results_quali.txt results_C1_1.txt results_C1_2.txt 6 16 c > final_prediction.txt

With the last script one can check the final qualitative results against a reference dataset.
python match_standalone.py -n collins2007.txt CYC08.txt results_filtered.txt

II. Supplementary Figures

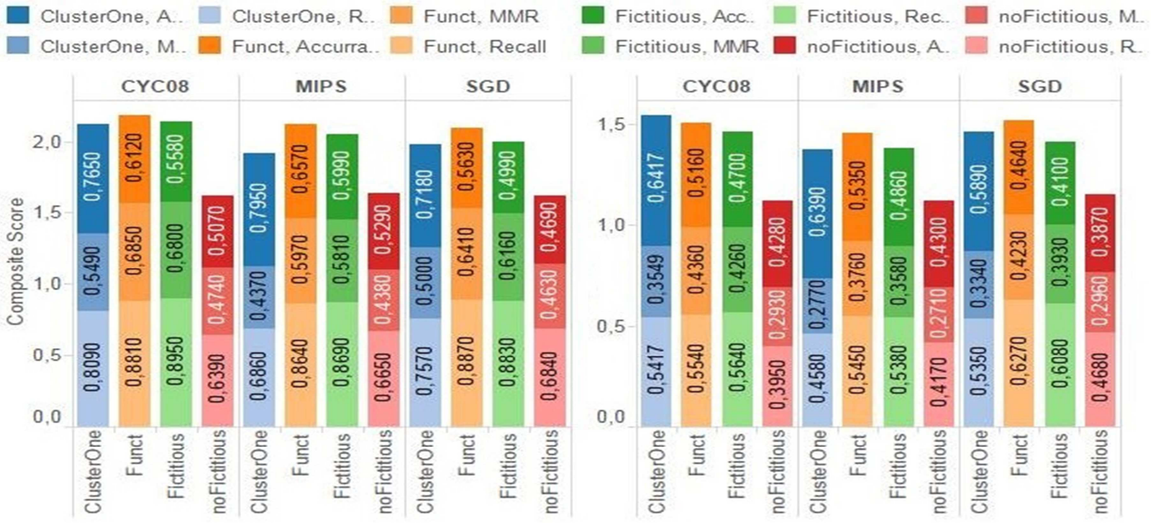

Figure 1. **Performance of fictitious binding strategies**. Results from one SiComPre simulation compared with predictions of clusterOne with complex sizes greater or equal 2 and using the Collins PPI dataset and checking against the reference complex sets from three sources (CYC08, MIPS (2012) and SGD). The *Function* strategy greatly outperforms the other binding strategies. In the left panel reference complexes are removed if their proteins were not found in the initial PPI, in the right panel we kept the whole reference dataset.

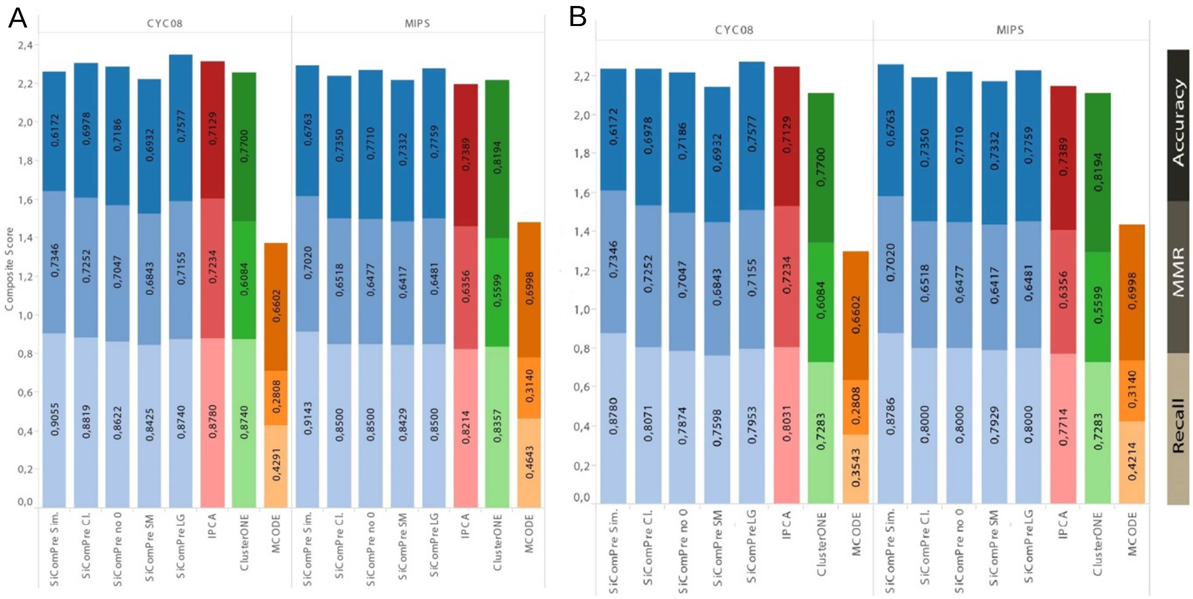

Figure 2. Composite score of different protein complex prediction methods tested against the CYC08 (left) and MIPS (right) budding yeast reference datasets of protein complexes. In this figure we show the performance of each step of SiComPre together with three earlier methods. **a.** composite scores reached by the various methods against the two datasets. SiComPre Sim is the set of complexes after the simulation, SiComPre CL. after the clustering, "no 0" is the set of complexes removing those which have 0 abundance, SiComPre SM after applying the strategy to optimize the f-score (dropping out small size low abundance complexes) and SiComPre LG after applying the strategy to optimize the composite score (dropping out large size low abundance complexes). After the clustering step SiComPre already gives better scores than any other methods, but the LG filtering to optimize the composite score increases the scores even more. **b.**

composite score of the different methods with the *updated recall* measure (calculated according the best matching complex, not considering any other matched complexes, see section 4.2 for details). In the case of MIPS all the SiComPre variants are better than any other methods, while for CYC08 SiComPre is always better than clusterOne and MCODE, while IPCA performs similarly to SiComPre with SiComPre-LG performing the best.

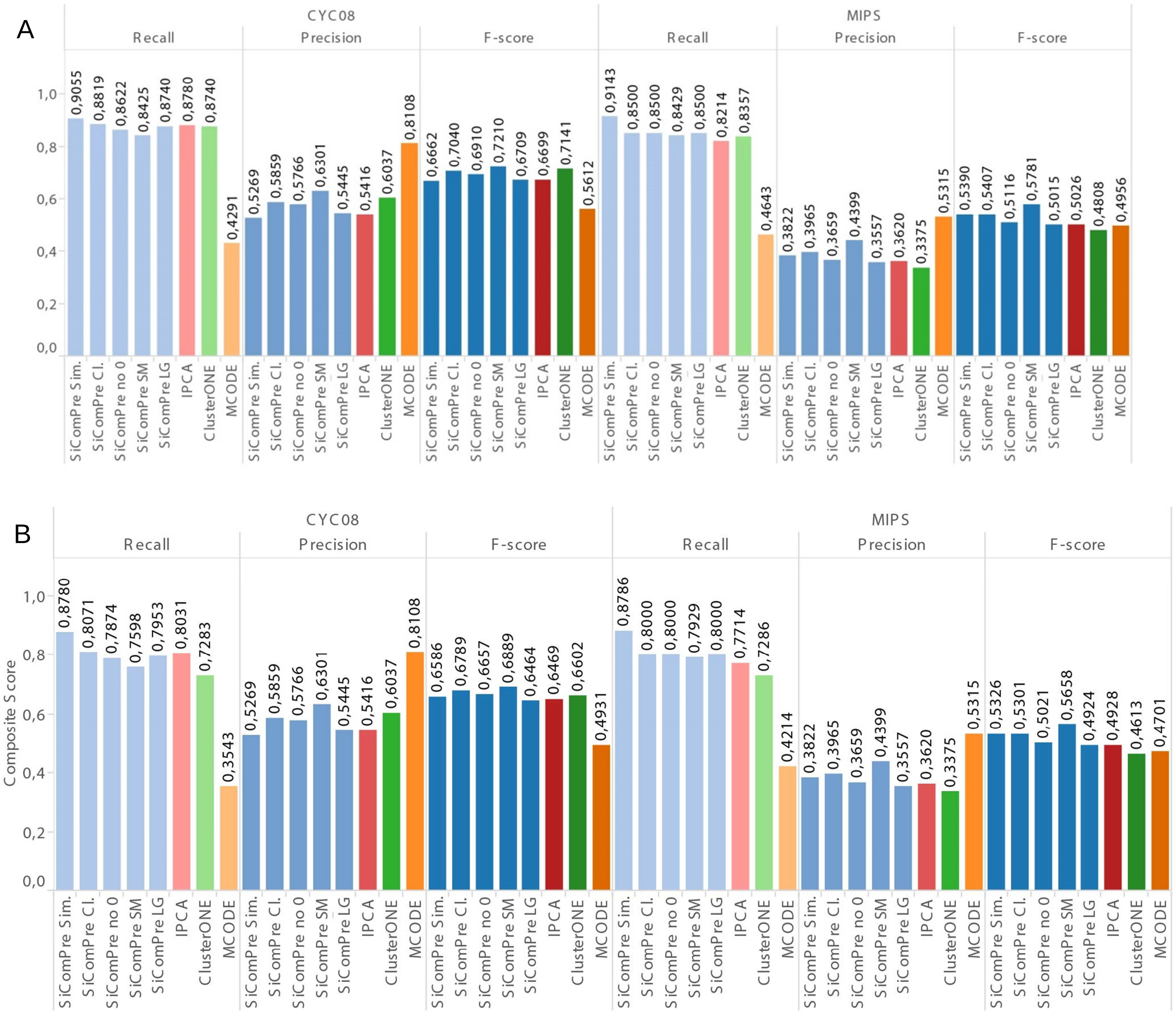

Figure 3. **F- score of different protein complex prediction methods tested against the CYC08 (left) and MIPS (right) budding yeast reference datasets of protein complexes.** Same as supplementary figure 3, but in this case we tested all methods by the f-score scoring system. The F-score (right column) is the harmonic mean of recall (left column) and precision (central column). Refer to Supplementary Figure 3 and "Qualitative results" section for the description of the strategies name. Considering the set of complexes after the clustering and after the optimization for the f-score only clusterOne against CYC08 dataset has better results, but, as showed in Supplementary Figure 1, it has a lower composite score. While our set of complexes after the optimization for the f-score have always better results. **b.** F-score according the *updated*

recall definition (calculated according the best matching complex, not considering any other matched complexes, see section 4.2 for details). In both reference dataset SiComPre have a better f-score than ClusterOne, IPCA and MCODE[5,29].

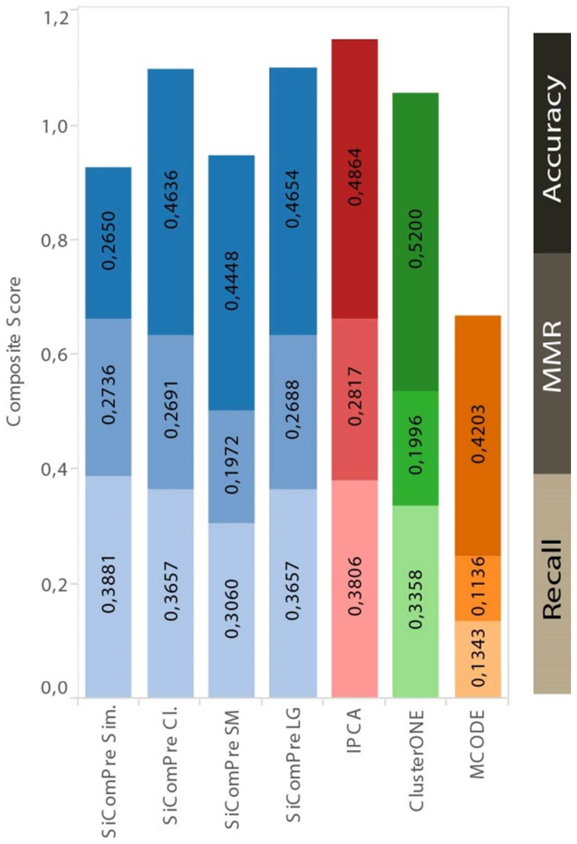

Figure 4. **Composite score of different methods with reference to the reduced human reference dataset of protein complexes.** Here we tested SiComPre and other earlier protein complex prediction methods on the human data against the reduced reference set from CORUM (redundancies and small size complexes removed, following Havugimana et al.[15]). In this test IPCA outperforms SiComPre, but IPCA predicts more complexes than SiComPre with a huge redundancy between them. Still for MMR and Recall SiComPre Sim gives the highest scores. For SiComPre version notations see supplementary Figure S3.

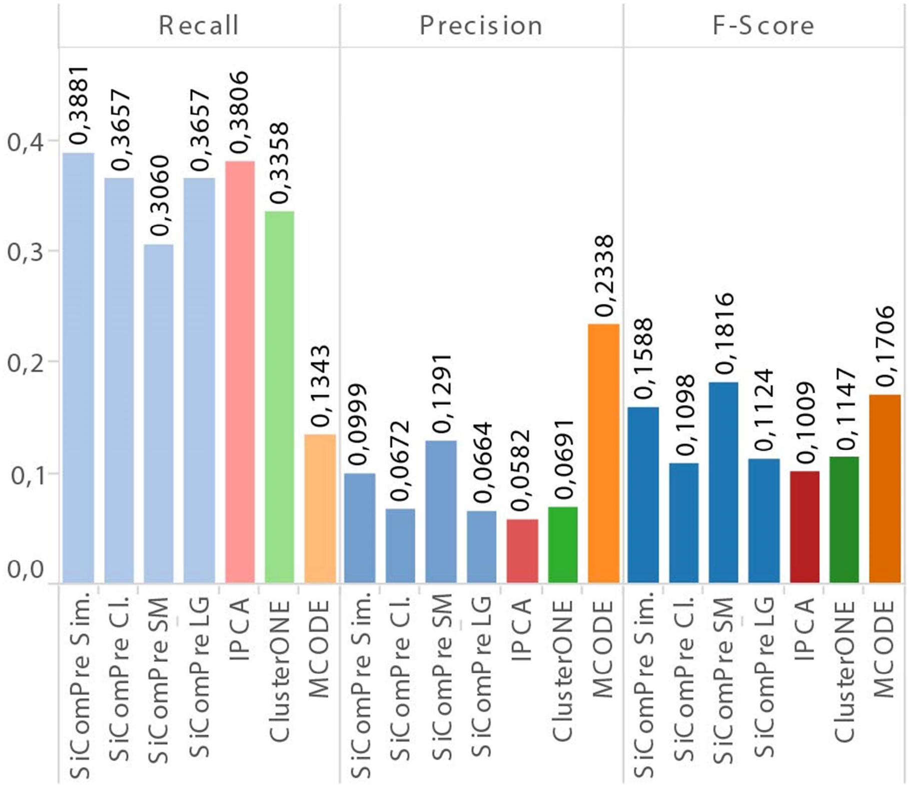

Figure 5. F- score of different methods with reference to the reduced human reference dataset of protein complexes. Similar to supplementary figure 5, but methods are measured against by the f-score system. The recall of SiComPre Sim is the highest while the –SM strategy to optimize f-score leads to the high precision and hence to the highest f-score. MCODE has a very high precision on a low number of complexes causing a poor recall.

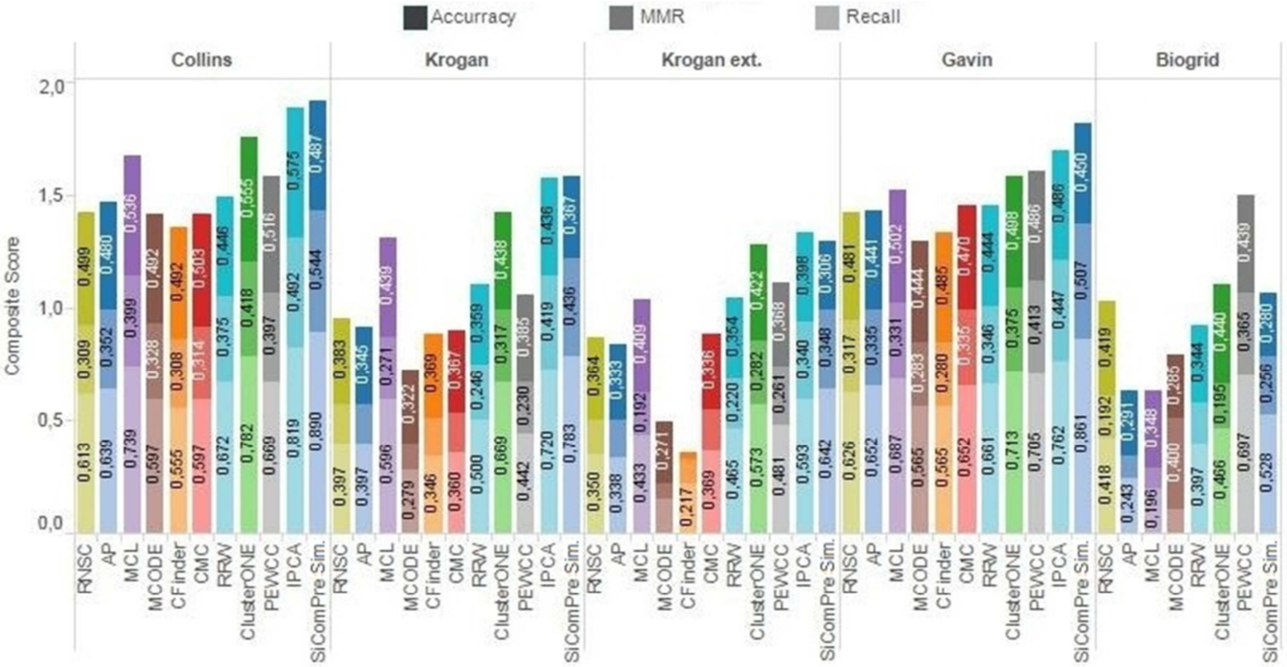

Figure 6. Composite scores based on various source data on yeast PPI by several existing methods compared tothat of SiComPre-SIM. Here, we are considering protein complexes of size >=3 and <= 100. Reference complexes are retrieved from MIPS, PPI datasets are Collins [10], Krogan, Krogan extended [30], Gavin[31] and Biogrid [32]. Results of other methods are copied from ClusterOne study, except for ClusterOne [12], PEWCC [14] and IPCA[9]. In this situation SiComPre can outperform every other method, except when using the Biogrid PPI, where PEWCC (an algorithm that can deal with high level of noise) and with Krogan extended where IPCA performs the best on the larger list of PPIs.

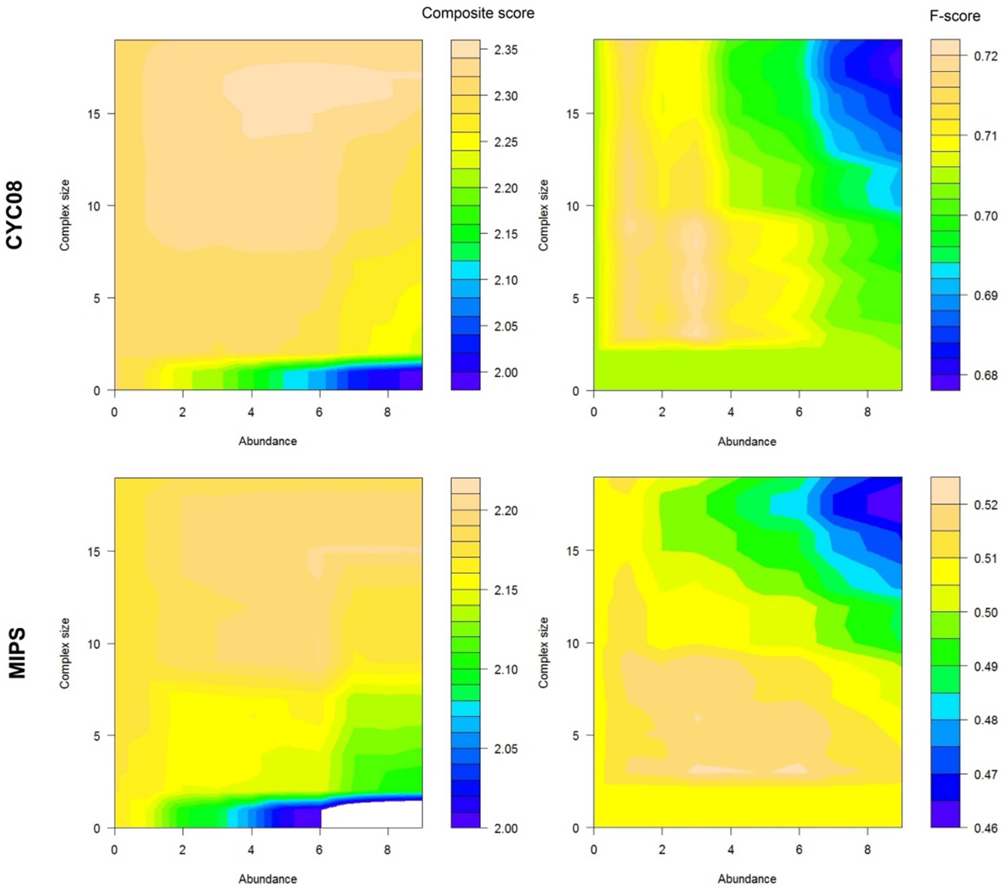

Figure 7. Optimizing *f-score* and *composite score* by removing large or small size low abundance complexes. We identified two different formulas to optimize the two quality measurment scores: i. by removing large size complexes with low abundance (left column) and ii. removing small size complexes with low abundance (right column). We saw that the first strategy leads to a higher composite score (see color code), while the second one achieve a better f-score. In the text we refer to these strategies as LG and SM respectively. In the left column we removed low abundance large size complezes, more specifically all complexes that do not satisfy the formula abundance >= x or complex size <= y. It is possible to observe that the optimal composite score for x and y are respectively 6 and 16. Following this all complexes with size greater than 16 and with abundance lower than 6 were removed from the predictions in the case of SiComPre-LG. The composite score is basically identical in the whole region (4-7, 15-17) when measured against two different yeast reference databases, thus we used the same values in the human SiComPre-LG predictions. The right column shows how f-score could be optimized by removing small size complexes with low abundance. Similar as above the formula this time we kept only complexes with abundance >= x or complex size >= y, the optimal value for x and y are both 3 and 3, again the reference database is not changing this value.. We have also checked *f-score* variation with the -LG strategy and *composite score* variation with the -SM strategy, but they did not show any imporvements (not shown).

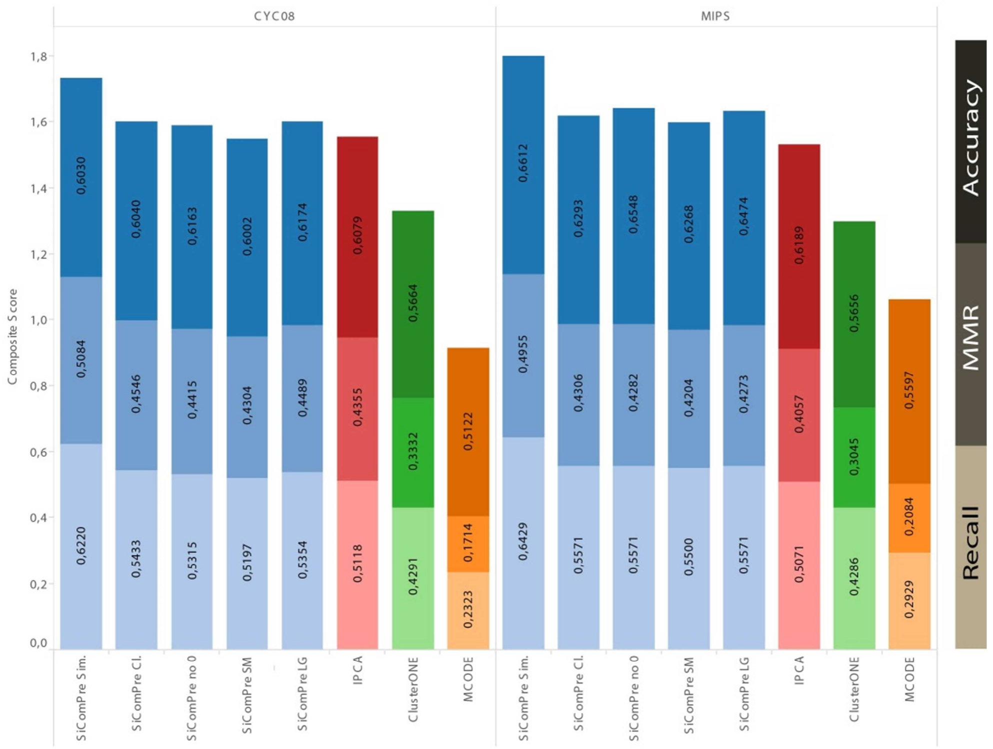

Figure 8. Composite score of various methods after removing non-matching compartment complexes with reference to CYC08 (left) and MIPS (right) yeast databases. Similar to supplementary figure 3, but in this case the composite score is computed after removing all complexes which have proteins that are not localized in the same compartment and do not contain membrane bound proteins. Basically, we removed every predicted complex that is not consistent with compartments data[5,29]. In this scenario every variant of SiComPre outperform any other methods.

III. Supplementary Tables Description

Supplementary Table S1: SiComPre predicted budding yeast protein complexes together with their predicted abundances

Supplementary Table S2: SiComPre predicted human protein complexes together with their predicted abundances

These tables are provided as separate Excel files. They were generated by the scripts generateCSV.py and generateCSVhuman.py found in **File S1.**

The column headings are abbreviations for:

- **ID**: ID of the refined complex.
- **RC Size**: The number of proteins contained in the refined complex.
- **List of proteins**: List of proteins contained in the refined complex (qualitative prediction).
- **Quantitative prediction**: the predicted average abundance of the complex, that is the number of simulated complexes associated to this refined complex. This value is the average between two simulations. It is important to remember that this value should be raised to the square, since in our simulations we considered only the square root of protein complexes.
- **Abundance Sim 1**: as above, but this is the abundance predicted by the first simulation. Note that this value is based on a prediction that use the square root of protein abundance, thus one should calculate the square of this value in order to get the true prediction. **Abundance Sim 2**: the abundance predicted by the second simulations. Note that this value is based on a prediction that use the square root of protein abundance, thus one should calculate the square of this value in order to get the true prediction.
- **Average size of simulated complexes**: The average size of simulated complexes associated to this refined complex.
- **Best matching reference complex**: the reference complex in CYC08 that has the highest overlap score with the refined complex.
- **Matching thr**: The overlap score between reference complex and refined complex.
- **Size of reference complex**: Number of proteins the CYC08 reference complex contains.
- **Fraction of fictitious domains**: The fraction of fictitious domains involved in the complex.
- **Functional enrichment**: Functional enrichment calculated with hypergeometric function, without any correction (low (<0.005 p-values mean the complex contains proteins with the given annotation in higher proportion than randomly would be expected). For yeast we list MIPS functions, while for human GO annotations.

Supplementary Table S3: SiComPre predictions on the effect of bortezomib on human protein complexes

This table is provided as separate Excel files.

The column headings mean (see Supplementary text Section 7 above for details):

Sheet 1(Normal vs. Bortezomib)

- **idref**: ID of the refined complex in normal condition.
- **Size**: The number of proteins contained in the refined complex (normal condition).
- **List of proteins in normal condition**: List of proteins contained in the refined complex in normal condition (qualitative prediction).
- **Quantitative prediction**: the predicted average abundance of the complex in normal condition, that is the number of simulated complexes associated to this refined complex. This value is the average between three simulations. It is important to remember that this value should be raised to the square, since in our simulations we considered only the square root of protein complexes.
- **Std Dev**: standard deviation of protein complex abundance over three runs.
- **List of proteins with bortezomib**: list of proteins contained in the refined complex retrieved from the simulations with Bortezomib that has the best matching with the corresponding normal condition refined complex.
- **Matching between new and old complex**: overlap score between refined complex in normal condition and after Bortezomib addition.
- **Quantitative prediction with bortezomib**: the predicted average abundance of the complex after the addition of Bortezomib (see quantitative prediction).
- **Std Dev with bortezomib:** standard deviation of protein complex abundance over three runs after Bortezomib addition.
- **T**: This value represents the statistical correlation between the observation of the complex in normal condition and the one after Bortezomib addition. To measure the correlation we used a t-test and the critical value considering 3 simulation runs and a probability of 0.01 is 5.84. Therefore, every complex above this threshold can be considered as a perturbation induced by the drug.
- **Best matching reference complex with normal condition**: the reference complex in CYC08 that has the highest overlap score with the normal condition refined complex.
- **Matching thr**: The overlap score between reference complex and normal condition refined complex.
- **Size of reference complex**: how many proteins CYC08 reference complex contains.
- **Best matching reference complex with bortezomib**: the reference complex in CYC08 that has the highest overlap score with the normal condition refined complex.
- **Matching thr**: The overlap score between reference complex and refined complex with bortezomib.
- **Size of reference complex**: how many proteins CYC08 reference complex contains.
- **Functional enrichment**: Functional enrichment calculated with hypergeometric function, without any correction (low (<0.005 p-values mean the complex contains proteins with the given annotation in higher proportion than randomly would be expected). For yeast we list MIPS functions, while for human GO annotations.

Sheet 2 (New Complexes): refers to description of table 1 and 2

Supplementary Table 5: Predictions of the fraction of unbound proteins by SiComPre simulations of the yeast and human data in Microsoft Excel format.

This table is provided as separate Excel files.

The column headings mean (see Supplementary text Section 7 above for details):

Sheet 1(Human) and Sheet 2(Yeast)

- **Protein**: ID of the protein

- **Fraction of free subunits**: fraction of subunits that at the end of simulation are not bounded to any other protein
- **Abundance:** abundance of the protein in the protein abundance dataset
- **# Interactions in the final model:** number of interactions in the final model in which the protein is involved

IV. Tables

Table 1. Protein domains interacting with Brotezomib.

List of protein domains identified with domain enrichment from the list of protein interacting with Bortezomib [18]. Analysis performed by the DAVID tool [19].

Pfam Accession	Name
PF00012	Hsp70 protein
PF00070	Pyridine nucleotide-disulphide oxidoreductase
PF00149	Calcineurin-like phosphoesterase
PF00227	Proteasome subunit
PF01851	Proteasome/cyclosome repeat
PF07992	Pyridine nucleotide-disulphide oxidoreductase
PF10584	Proteasome subunit A N-terminal signature

V. References

1. Baeten JCM (2005) A brief history of process algebra. Theoretical Computer Science 335: 131-146.
2. Dematté L, Priami C, Romanel A (2008) Modelling and simulation of biological processes in BlenX. ACM SIGMETRICS Performance Evaluation Review 35: 32-39.
3. Gillespie DT (2001) Approximate accelerated stochastic simulation of chemically reacting systems. The Journal of Chemical Physics 115: 1716.
4. Letunic I, Doerks T, Bork P (2012) SMART 7: recent updates to the protein domain annotation resource. Nucleic Acids Res 40: D302-305.
5. Mewes H-W, Frishman D, Mayer KF, Münsterkötter M, Noubibou O, et al. (2006) MIPS: analysis and annotation of proteins from whole genomes in 2005. Nucleic Acids Res 34: D169-D172.
6. Gillespie DT (2007) Stochastic simulation of chemical kinetics. Annu Rev Phys Chem 58: 35-55.
7. Dematte L, Prandi D (2010) GPU computing for systems biology. Brief Bioinform 11: 323-333.
8. Rodriguez JV, Kaandorp JA, Dobrzynski M, Blom JG (2006) Spatial stochastic modelling of the phosphoenolpyruvate-dependent phosphotransferase (PTS) pathway in Escherichia coli. Bioinformatics 22: 1895-1901.
9. Li M, Chen JE, Wang JX, Hu B, Chen G (2008) Modifying the DPClus algorithm for identifying protein complexes based on new topological structures. BMC Bioinformatics 9: 398.
10. Collins SR, Kemmeren P, Zhao X-C, Greenblatt JF, Spencer F, et al. (2007) Toward a comprehensive atlas of the physical interactome of Saccharomyces cerevisiae. Molecular & Cellular Proteomics 6: 439-450.
11. Bader GD, Hogue CW (2003) An automated method for finding molecular complexes in large protein interaction networks. BMC Bioinformatics 4: 2.
12. Nepusz T, Yu H, Paccanaro A (2012) Detecting overlapping protein complexes in protein-protein interaction networks. Nature Methods 9: 471-472.
13. Brohee S, van Helden J (2006) Evaluation of clustering algorithms for protein-protein interaction networks. BMC Bioinformatics 7: 488.
14. Zaki N, Efimov D, Berengueres J (2013) Protein complex detection using interaction reliability assessment and weighted clustering coefficient. BMC Bioinformatics 14.
15. Havugimana Pierre C, Hart GT, Nepusz T, Yang H, Turinsky Andrei L, et al. (2012) A Census of Human Soluble Protein Complexes. Cell 150: 1068-1081.
16. Mewes HW, Amid C, Arnold R, Frishman D, Güldener U, et al. (2004) MIPS : analysis and annotation of proteins from whole genomes. Nucleic Acids Res 32: D41-D44.
17. Ashburner M, Ball CA, Blake JA, Botstein D, Butler H, et al. (2000) Gene ontology: tool for the unification of biology. The Gene Ontology Consortium. Nat Genet 25: 25-29.
18. Kuhn M, Szklarczyk D, Pletscher-Frankild S, Blicher TH, von Mering C, et al. (2014) STITCH 4: integration of protein-chemical interactions with user data. Nucleic Acids Res 42: D401-407.
19. Huang da W, Sherman BT, Lempicki RA (2009) Systematic and integrative analysis of large gene lists using DAVID bioinformatics resources. Nat Protoc 4: 44-57.
20. Huang da W, Sherman BT, Lempicki RA (2009) Bioinformatics enrichment tools: paths toward the comprehensive functional analysis of large gene lists. Nucleic Acids Res 37: 1-13.
21. Zhang H-M, Chen H, Liu W, Liu H, Gong J, et al. (2012) AnimalTFDB: a comprehensive animal transcription factor database. Nucleic Acids Research 40: D144-D149.
22. McKusick-Nathans Institute of Genetic Medicine JHUB, MD) Online Mendelian Inheritance in Man, OMIM®.
23. Fraser HB, Plotkin JB (2007) Using protein complexes to predict phenotypic effects of gene mutation. Genome Biology.
24. Bantscheff M, Hopf C, Savitski MM, Dittmann A, Grandi P, et al. (2011) Chemoproteomics profiling of HDAC inhibitors reveals selective targeting of HDAC complexes. Nat Biotechnol 29: 255-265.
25. Kolker E, Higdon R, Haynes W, Welch D, Broomall W, et al. (2012) MOPED: Model Organism Protein Expression Database. Nucleic Acids Res 40: D1093-1099.
26. Uhlen M, Fagerberg L, Hallstrom BM, Lindskog C, Oksvold P, et al. (2015) Proteomics. Tissue-based map of the human proteome. Science 347: 1260419.

27. Mehdi AM, Patrick R, Bailey TL, Boden M (2014) Predicting the dynamics of protein abundance. Mol Cell Proteomics 13: 1330-1340.
28. Babu M, Vlasblom J, Pu S, Guo X, Graham C, et al. (2012) Interaction landscape of membrane-protein complexes in Saccharomyces cerevisiae. Nature 489: 585-589.
29. Binder JX, Pletscher-Frankild S, Tsafou K, Stolte C, O'Donoghue SI, et al. (2014) COMPARTMENTS: unification and visualization of protein subcellular localization evidence. Database (Oxford) 2014: bau012.
30. Krogan NJ, Cagney G, Yu H, Zhong G, Guo X, et al. (2006) Global landscape of protein complexes in the yeast Saccharomyces cerevisiae. Nature 440: 637-643.
31. Gavin AC, Aloy P, Grandi P, Krause R, Boesche M, et al. (2006) Proteome survey reveals modularity of the yeast cell machinery. Nature 440: 631-636.
32. Chatr-Aryamontri A, Breitkreutz BJ, Heinicke S, Boucher L, Winter A, et al. (2013) The BioGRID interaction database: 2013 update. Nucleic Acids Res 41: D816-823.
33. Ghaemmaghami S, Huh W-K, Bower K, Howson RW, Belle A, et al. (2003) Global analysis of protein expression in yeast. Nature 425: 737-741.
34. Lorch JH, Thomas TO, Schmoll H-J (2007) Bortezomib Inhibits Cell-Cell Adhesion and Cell Migration and Enhances Epidermal Growth Factor Receptor Inhibitor–Induced Cell Death in Squamous Cell Cancer. Cancer Research 67: 727-734.
35. Poulaki V, Mitsiades CS, Kotoula V, Negri J, McMillin D, et al. (2007) The Proteasome Inhibitor Bortezomib Induces Apoptosis in Human Retinoblastoma Cell Lines In Vitro. Investigative Ophthalmology & Visual Science 48: 4706-4719.
36. Khandros E, Thom CS, D'Souza J, Weiss MJ (2012) Integrated protein quality-control pathways regulate free α-globin in murine β-thalassemia. 5265-5275 p.
37. Rossi A, Riccio A, Coccia M, Trotta E, La Frazia S, et al. (2014) The Proteasome Inhibitor Bortezomib Is a Potent Inducer of Zinc Finger AN1-type Domain 2a Gene Expression: ROLE OF HEAT SHOCK FACTOR 1 (HSF1)-HEAT SHOCK FACTOR 2 (HSF2) HETEROCOMPLEXES. Journal of Biological Chemistry 289: 12705-12715.
38. Wacker SA, Houghtaling BR, Elemento O, Kapoor TM (2012) Using transcriptome sequencing to identify mechanisms of drug action and resistance. Nat Chem Biol 8: 235-237.
39. Maynadier M, Shi J, Vaillant O, Gary-Bobo M, Basile I, et al. (2012) Roles of Estrogen Receptor and p21Waf1 in Bortezomib-Induced Growth Inhibition in Human Breast Cancer Cells. Molecular Cancer Research 10: 1473-1481.
