# Supplementary material for: Qualitative and Quantitative Protein Complex Prediction Through Proteome-Wide Simulations
Source: PLoS Comput Biol. 2015 Oct 22;11(10):e1004424. doi: 10.1371/journal.pcbi.1004424 (PMC4619657; doi:10.1371/journal.pcbi.1004424)
Supplement: S1 Fig — (DOCX) [file pcbi.1004424.s008.docx]

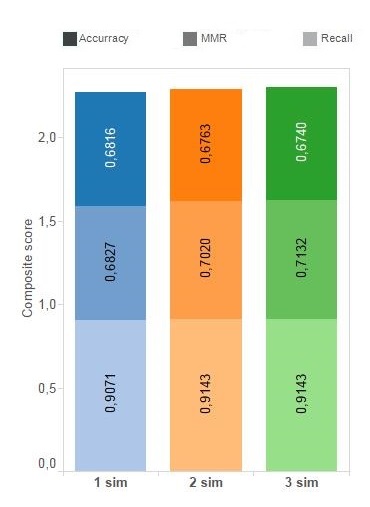


**S1 Fig.** **Effect of the number of considered simulations on qualitative predictions**. The increase in the number of simulation runs considered in SiComPre shows only a slight improvement of MMR and recall, but the accuracy decreases after two runs. This was checked only for the yeast data as the annotation of human complexes is much sparser.
