# Supplementary material for: Qualitative and Quantitative Protein Complex Prediction Through Proteome-Wide Simulations
Source: PLoS Comput Biol. 2015 Oct 22;11(10):e1004424. doi: 10.1371/journal.pcbi.1004424 (PMC4619657; doi:10.1371/journal.pcbi.1004424)
Supplement: S2 Fig — (DOCX) [file pcbi.1004424.s009.docx]

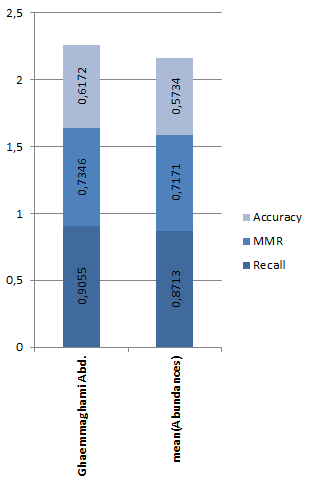


**S2 Fig. Composite scores of protein complex qualitative predictions after two simulations with actual (left) and average (right) protein abundances.** The left column results are based on simulations running with protein abundances from Ghaemmaghami et al.[[33](#_ENREF_33)] (same as SiComPre SIM. on Supplementary Figure S3). The second set of simulations were performed setting protein abundance of each protein to the mean of protein abundances in Ghaemmaghami et al. The use of experimental protein abundances improved the composite score by 0.1, while also increasing all of its sub-scores.
